# Biosurfactant derived from probiotic *Lactobacillus acidophilus* exhibits broad-spectrum antibiofilm activity and inhibits the quorum sensing-regulated virulence

**DOI:** 10.17305/bb.2023.9324

**Published:** 2023-12-01

**Authors:** Mohd Adnan, Arif Jamal Siddiqui, Emira Noumi, Syed Amir Ashraf, Amir Mahgoub Awadelkareem, Sibte Hadi, Mejdi Snoussi, Riadh Badraoui, Fevzi Bardakci, Manojkumar Sachidanandan, Mitesh Patel

**Affiliations:** 1 Department of Biology, College of Science, University of Ha’il, Ha’il, Saudi Arabia; 2 Department of Clinical Nutrition, College of Applied Medial Sciences, University of Ha’il, Ha’il, Saudi Arabia; 3 Department of Forensic Sciences, Naif Arab University for Security Sciences, Riyadh, Saudi Arabia; 4 Department of Oral Radiology, College of Dentistry, University of Ha’il, Ha’il, Saudi Arabia; 5 Department of Biotechnology, Parul Institute of Applied Sciences and Centre of Research for Development, Parul University, Vadodara, India

**Keywords:** Biosurfactant, *Lactobacillus acidophilus*, antibiofilm, quorum sensing (QS), virulence

## Abstract

Antimicrobial resistance by pathogenic bacteria has become a global risk to human health in recent years. The most promising approach to combating antimicrobial resistance is to target virulent traits of bacteria. In the present study, a biosurfactant derived from the probiotic strain *Lactobacillus acidophilus* was tested against three Gram-negative bacteria to evaluate its inhibitory potential on their biofilms, and whether it affected the virulence factors controlled by quorum sensing (QS). A reduction in the virulence factors of *Chromobacterium violaceum* (violacein production), *Serratia marcescens* (prodigiosin production), and *Pseudomonas aeruginosa* (pyocyanin, total protease, LasB elastase, and LasA protease production) was observed at different sub-minimum inhibitory concentration (MIC) concentrations in a dose-dependent manner. Biofilm development was reduced by 65.76%, 70.64%, and 58.12% at the highest sub-MIC levels for *C. violaceum*, *P. aeruginosa*, and *S. marcescens*, respectively. Biofilm formation on glass surfaces exhibited significant reduction, with less bacterial aggregation and reduced formation of extracellular polymeric materials. Additionally, swimming motility and exopolysaccharides (EPS) production were shown to be reduced in the presence of the *L. acidophilus*-derived biosurfactant. Furthermore, molecular docking analysis performed on compounds identified through gas chromatography–mass spectrometry (GC-MS) analysis of QS and biofilm proteins yielded further insights into the mechanism underlying the anti-QS activity. Therefore, the present study has clearly demonstrated that a biosurfactant derived from *L. acidophilus* can significantly inhibit virulence factors of Gram-negative pathogenic bacteria. This could provide an effective method to inhibit the formation of biofilms and QS in Gram-negative bacteria.

## Introduction

Infectious diseases are largely caused by bacterial infections, and recent advancements in research indicate that there is a growing demand in research for natural products. These products have been demonstrated to be able to cure diseases of a severe and debilitating nature [1]. Numerous bioactive metabolites can be found in different types of natural products, which serve as therapeutic agents and are responsible for curing a wide range of diseases [2]. Typically, bioactive compounds are derived from plants, microbes, and animals, which naturally protect humans from various diseases, making them potential drug candidates. A wide range of bioactive compounds have been identified that have powerful antioxidative, cytotoxic, antimicrobial, and anti-inflammatory properties [3]. At present, some pharmaceutical industries are searching for new antimicrobial and antibiofilm agents derived from natural compounds [4].

A quorum sensing (QS) process is a way for bacteria to communicate by detecting concentrations of signaling molecules in their environment. A small signaling molecule known as an autoinducer is secreted by bacteria, which is diffused out of the cells, and accumulates in the surrounding environment [5]. As a bacterial population grows, the concentration of autoinducers increases, and once a threshold concentration is reached, bacteria are able to sense one another and respond. As a result of this process, bacteria are capable of forming biofilms, increasing their virulence, and developing resistance to antibiotics [6]. Using QS, pathogenic bacteria can be eliminated or inhibited by exchanging information between cells and controlling gene expression to control cell density. There has been considerable evidence indicating that QS occurs as a result of extracellular signaling molecules, known as autoinducers, which are produced, detected, and responded to by pathogenic bacteria. Various bacterial virulence factors are regulated by QS, including adhesion to different surfaces, biofilm formation, and synthesis of an extracellular matrix that plays an important role in the biofilm development [7].

In Gram-negative pathogenic bacteria, such as *Pseudomonas aeruginosa* (*P. aeruginosa*), *Chromobacterium violaceum* (*C. violaceum*), and *Serratia marcescens* (*S. marcescens*), different QS signals are effective in infecting individuals, specifically those with weak immune systems. These bacterial pathogens are challenging to eliminate because of their high pattern of resistance to antimicrobial agents and the synthesis of many virulence factors [8]. Currently, several antimicrobial agents are available to treat invasive microbial infections including chloramphenicol, rifampicin, tetracyclines, temocillin, polyenes, fluconazole, polymyxins, trimethoprim-sulfamethoxazole, voriconazole and nitrofurantoin, etc. Nevertheless, pathogenic bacteria are continuously developing resistance to these medications [9]. As a result of this rationale, researchers are increasingly exploring the potential of natural products as next-generation therapeutics and anti-pathogenic products [10].

There are numerous types of bacteria that are capable of producing surface-active compounds that have biomedical or biotechnological applications. The biosurfactants are surface-active compounds that have polar and non-polar characteristics [11]. As a result of their high biodegradability, environmental, and eco-friendly properties, they can be employed in a variety of fields. Many of them have low toxicity toward humans and can maintain their activity under extreme pH and temperature conditions [12]. They play an important role in maintaining microbial homeostasis, primarily in the mouth and vaginal cavity [13]. Moreover, surface-active compounds have been reported for antibacterial properties, antifungal, antiviral, anticancer, anti-adhesive, and anti-inflammatory activities [14–19]. Accordingly, the objective of the current study was to evaluate the biosurfactant production and extraction from the lactic acid bacteria *L. acidophilus,* as well as to assess their antibacterial, antibiofilm, and anti-QS activities against pathogenic Gram-negative bacteria known to form biofilms.

## Materials and methods

### Bacterial strains and growth conditions

The pathogenic bacterial strains *P. aeruginosa* MTCC-741, *C. violaceum* MTCC-2656, *S. marcescens* MTCC-97 and lactic acid bacteria strain, *L. acidophilus* MTCC-10307 were collected from the Microbial Type Culture Collection (IMTECH, Chandigarh, India). For the growth and maintenance of the lactic acid bacteria strain, the De Man, Rogosa and Sharpe (MRS) agar plate (HiMedia^®^, Mumbai, India) was used, whereas, for *P. aeruginosa*, Luria-Bertani agar (LB, HiMedia^®^, Mumbai, India) was used. Both bacterial strains were stored at 4 ^∘^C for further use.

### Biosurfactant assays

The log phase culture of *L. acidophilus* was inoculated into MRS-Lac broth (glucose was replaced with lactose from the media composition) and incubated for 72 h at 30 ^∘^C. After incubation, centrifugation was performed for 10 min at 10,000 rpm and 4 ^∘^C to collect the supernatant. From the obtained supernatant, biosurfactant production was confirmed by performing various methods.

#### Oil displacement assay

The method described by Joe et al. [20] was used to perform the oil displacement assay. A Petri dish was filled with distilled water (50 mL) and crude oil (2 mL) was added so that it was evenly distributed on the water surface. After that, 500 µL of culture supernatant was carefully spotted on the center of the oily layer surface. Then, the diameter of the clear zones was measured after 30 s. As a negative control, distilled water was used.

#### Drop collapse assay

The method described by Płaza et al. [21] was used to perform the drop collapse assay. To observe the drop collapse activity, culture supernatant (50 µL) was placed on parafilm. The collapse of the drop was considered a positive result, indicating the presence of biosurfactants in the solution.

#### Emulsification assay

The method described by Satpute et al. [22] was used to perform the emulsification assay. After mixing equal volumes of the culture supernatant with olive oil, petrol, and kerosene for 2 min using vortex, the mixture was allowed to stand for 24 h. In order to calculate emulsification index (%E24), the following equation was used.


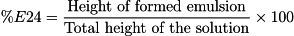


#### Measurements of surface tension

A tensiometer (K11, Kruss, Hamburg, Germany) was used to measure surface tension. Before use, the tensiometer was calibrated with distilled water (72 mN/m). Approximately 20 mL of the culture supernatant was placed in a sterile beaker and placed on the sample table. As part of the procedure, the height of the sample pool was maintained in a way that the platinum ring, which was hanging from the balance hook, was immersed beneath the liquid surface of the sample during the equilibration process. It was then lifted up. When the ring was pulled through the liquid surface by a microbalance, the force applied to the ring was recorded. A platinum ring that drops below the liquid level was displayed as a value representing the surface tension of the sample at that point. As a control, non-inoculated medium was used [23].

### Biosurfactant production and extraction

*L. acidophilus* active culture (1%) was added to MRS-Lac (500 mL) broth and incubated for 72 h at 37 ^∘^C without shaking to produce crude biosurfactants. To extract biosurfactants, the culture broth was centrifugated at 10,000 rpm for 10 min at 4 ^∘^C to get culture supernatant. By adding 6N HCl to the supernatant, the pH was adjusted to 2 and the supernatant was stored at 4 ^∘^C for the next day. Ethyl acetate was used to continuously elute the biosurfactant from the refrigerated supernatant at room temperature. The ethyl acetate and supernatant were thoroughly mixed in a 1:1 mixer and then left stationary so that phase separation could take place. The organic phase was then collected, transferred to a rotary evaporator, and then evaporated under reduced pressure at 40 ^∘^C to yield a dark honey-colored viscous product. A gravimetric procedure was carried out to determine the amount of crude biosurfactant [24].

### Characterization of the extracted biosurfactant

The Fourier-transform infrared spectroscopy (FTIR) analysis (Bruker^®^, Billerica, MA, USA) was performed on the extracted crude biosurfactants to determine their chemical structure and components. The sample was directly used and IR spectrum was recorded from 400 to 4000 cm^−1^ wave range from with a resolution of 4 cm^−1^ [25].

### Antibacterial activity determination

Well diffusion/agar cup method was used to test the antibacterial potential of extracted biosurfactant against *C. violaceum*, *P. aeruginosa*, and *S. marcescens* [26]. The turbidity of the culture was adjusted with sterile saline solution after the bacterial culture was grown overnight at 37 ^∘^C in a fresh LB medium. A sterile cork borer was used to create wells in the plates after the culture was evenly distributed on the plates (100 µL). One well was inoculated with 60 µL of crude biosurfactant and incubated at 37 ^∘^C for 24 h. The zones of inhibition were observed the following day. Sterile water was used as a negative control and chloramphenicol as a positive control.

### Minimum inhibitory concentration determination

The determination of minimum inhibitory concentration (MIC) of *L. acidophilus*-extracted biosurfactant was carried out in 96-well plate by method reported previously [27]. Different concentrations of the crude biosurfactant (100 µL) ranging from 0.156 to 10 mg/mL together with active culture of *P. aeruginosa* (10^8^ CFU/mL) were added to a 96-well plate and incubated for 24 h at 37 ^∘^C. As the growth of bacteria in the plate was monitored after incubation, MIC value was calculated based on the concentration required to inhibit observable growth. As a negative control, media-containing wells were used, whereas positive controls used only inoculated bacteria in a well without biosurfactant.

### Antibiofilm assay

To determine the antibiofilm effect of *L. acidophilus*-derived biosurfactant, glass test tubes were used as hydrophilic surface [28]. Concisely, sterilized LB medium (3 mL) was transferred into the tubes containing 500 µL of extracted biosurfactants (sub-MICs) and active bacterial culture (1 mL). The tubes were then thoroughly mixed and incubated in a shaker incubator for 72 h at room temperature. After the incubation period, planktonic cells were removed, and the tubes were washed with the PBS. Later, crystal violet was used to stain the formed biofilm. Excess dye was removed by washing the tubes with PBS. Further, acetic acid was used for dissolving the stained biofilm, and absorbance was determined at 595 nm using a spectrophotometer. LB medium containing test bacterial strains was used as a control. Biofilm inhibition percentage was estimated using the following formula:


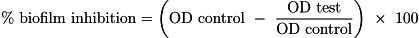


OD – optical density.

### Exopolysaccharide production determination

A ruthenium red staining assay was used to determine the ability of the extracted biosurfactant to inhibit the production of exopolysaccharide (EPS) matrix [29]. The active culture (100 µL) of the tested bacterial strains (10^8^ CFU/mL) and the extracted biosurfactant (sub-MICs) were incubated at 37 ^∘^C for 24 h. At the end of the incubation, the planktonic cells were removed, and the wells were washed with PBS (200 µL). To stain the biofilms formed by the adherent cells, 0.01% ruthenium red was added to each well. As a blank, ruthenium red was used (200 µL) to fill the wells, and then the wells were incubated at 37 ^∘^C for 60 min. Following that, a new microtiter plate was used to measure the absorbance at 450 nm of the liquid containing the residual stain. To calculate the amount of dye that has been fixed to biofilms, the following formula was used:

Abs_BF_ ═ Abs_B_ − Abs_S_

Abs_B _- absorbance of blank

Abs_S _- absorbance of residual stain collected from samples.

### Antibiofilm activity assessment by light microscopy

Biofilms formed by bacterial pathogens on glass coverslips were visualized using the method described by Musthafa et al. [30] with a few modifications. Coverslips were inserted in 6-well plates containing test cultures in LB supplemented with 0.2% glucose (10^8^ CFU/mL). Extracted biosurfactants (1/2 MIC) were added to the wells as a treatment. After 48 h of incubation at 37 ^∘^C, the glass coverslips containing the biofilms were gently detached and washed with PBS. The 0.1% crystal violet was used for biofilm staining, followed by observation under light microscope at 40× magnification (Axioscope A1, Zeiss, Jena, Germany).

### Violacein pigment production assessment in *C. violaceum*

According to standard procedure [31], quantitative assessment of violacein production was performed. With and without varying sub-MIC concentrations of the extracted biosurfactant, *C. violaceum* was grown at 30 ^∘^C for 18 h. Further, 1 mL of culture was centrifuged at 10,000 rpm for 5 min to separate the violacein from the bacterial cells. Cell pellet was then resuspended in DMSO (1 mL) to dissolve the pigment, followed by vigorous vortexing for 5 min. The suspension was then centrifuged again to spin down the bacterial debris. Absorbance of the supernatant was then measured at 585 nm using a UV-spectrophotometer (UV-2600, Shimadzu, Japan).

### Prodigiosin pigment production assessment in *S. marcescens*

According to the standard procedure [32], assessment of prodigiosin pigment production was performed using LB medium. Into the sterile LB medium, *S. marcescens* active culture was added with and without varying sub-MIC concentrations of the extracted biosurfactant and grown overnight at 30 ^∘^C. After incubation, the cell pellet was then collected by centrifuging 2 mL of the grown culture at 10,000 rpm for 10 min. Obtained cell pellet was then dissolved in acidified ethanol (4 mL 1 M HCl + 96 mL ethanol) at room temperature by vigorous shaking. Sample was then centrifuged again to remove the debris. Supernatant absorbance was measured at 534 nm using a UV-spectrophotometer (UV-2600, Shimadzu, Japan).

### Quorum sensing inhibitory activity determination in *P. aeruginosa*

#### Pyocyanin production quantitative analysis in *P. aeruginosa*

In the absence or presence of extracted biosurfactant, pyocyanin pigment production was determined from the supernatants of *P. aeruginosa* culture following the method described by Ugurlu et al. [33]. In the first step, 1.5 mL of the supernatant of *P. aeruginosa*, whether untreated or treated with sub-MIC concentrations, was first extracted with 3 mL of chloroform and then with 0.2 M HCl (700 µL). In the following step, the obtained solution was transferred to a glass cuvette, which was then used to determine the absorbance at 595 nm. To quantify the pyocyanin production, following formula has been used:


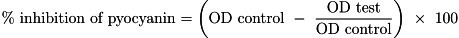


#### LasA staphylolytic assay

Using *P. aeruginosa* culture supernatant to lyse boiled *S. aureus* cells, LasA protease activity was determined [34]. First, the overnight grown culture of *S. aureus* (10^6^ CFU/mL) was centrifuged at 8000 rpm for 5 min. The obtained cell pellets were dissolved in 0.02 M Tris-HCl buffer (pH-8.5) and boiled for 10 min. It was then further diluted with 0.02 M Tris-HCl buffer to adjust OD of 0.8 at 595 nm. Thereafter, the supernatants of cell-free cultures of *P. aeruginosa* were added with diluted *S. aureus* suspension that was treated at sub-MIC levels or left untreated (in a 9:1 ratio). To determine percentage inhibition, readings were taken at 595 nm.

#### LasB elastase assay

An elastolytic activity measurement was performed following the procedure reported by Adonizio et al. [35]. First, *P. aeruginosa* culture was treated with crude biosurfactant (sub-MICs). After that, 900 µL of elastin Congo red buffer (100 mM Tris, 1.5 mM CaCl_2_, pH−7.5) containing 20 mg of elastin Congo red (Sigma^®^, Bengaluru, India) was added to the treated or control culture supernatant (Sigma^®^, Bengaluru, India) and incubated at 37 ^∘^C for 3 h. Afterward, centrifugation was performed to remove the insoluble components (elastin Congo red). Then, the absorbance of the supernatant was determined by spectrophotometric analysis at 495 nm. As a negative control, LB medium with or without crude biosurfactant was used.

### Azocasein assay for proteolytic activity

The procedure reported by Ugurlu et al. [33] was followed to determine the proteolytic activity in the supernatant of *P. aeruginosa* with (sub-MICs) or without treatment with extracted biosurfactant. The culture supernatant (150 µL) and 1 mL of 0.3% azocasein (dissolved in 0.05 M Tris-HCl and 0.5 mM CaCl_2_, pH−7.5) were mixed and incubated at 37 ^∘^C for 15 min. Then, 0.5 mL of 10% trichloroacetic acid was added to stop the reaction. After centrifugation of the sample, the absorbance was measured at 400 nm of the prepared sample at the end of process.

### Swarming motility assay

According to Packiavathy et al. [36], the swarming motility of *P. aeruginosa* and *S. marcescens* was measured. A swarming motility assay was performed with agar plates containing 1% tryptone, 0.5% NaCl, 0.3% agar, and 0.5% glucose with or without extracted biosurfactant (1/2 MIC). Plates were incubated for 24 h at 37 ^∘^C in the upright position.

### Gas chromatography-mass spectrophotometry analysis

In order to determine the number and type of components present in the extracted biosurfactants, gas chromatography–mass spectrometry (GC-MS) analysis was conducted using Shimadzu Nexis GC-2030 Gas Chromatograph (GC) in conjunction with a QP2020 NX Mass Spectrometer. To separate the sample, the column temperature was set to 50 ^∘^C for 3 min, further increased by 10 ^∘^C per min for 10 min until 270 ^∘^C was reached, and then was raised to 300 ^∘^C for 10 min before the separation was completed. The partially purified biosurfactant (0.1 g) was dissolved in methanol (100 µg/mL) and 10 µL of the sample was injected into the system where helium was used as the carrier gas. The obtained GC-MS peaks were compared with the NIST database in order to determine the probable composition of the crude extract [18].

### Molecular docking analysis

Molecular docking with AutoDock Vina was further used to investigate the mechanisms of extracted biosurfactant antibiofilm and anti-QS activity [37]. Using Open Babel 3.1.1, the three-dimensional (3D) structures of the GC-MS identified compounds were converted from .sdf to .pdb format. To obtain the best conformation, the ligand was made flexible with MGL Tools-1.5.7, and the coordinates were saved as .pdbqt. 3D crystal structures were downloaded for receptor proteins (LasI, EsaI, LasR, LasA, CviR, CviR′, PqsR, PilT, and PilY1) from Protein Data Bank. The crystal structure was modified by removing water molecules and adding hydrogen and Kollman charges. Protein coordinates were saved in .pdbqt format. PyMol and Discovery Studio were used to analyze docked complexes [38, 39].

## Results

### Biosurfactant production by *L. acidophilus*

The ability of *L. acidophilus* to produce biosurfactant was tested through a variety of qualitative and quantitative assays from the supernatant. Oil displacement and drop collapse assays are rapid methods for the selection of microbial biosurfactant producers. The results revealed that *L. acidophilus* was a good producer of biosurfactants. The emulsification capacity of *L. acidophilus* supernatant was estimated against different hydrocarbon substrates. The highest emulsification activity was obtained against olive oil (68.70%) compared with petrol (45.34%) and kerosene (35.68%). Furthermore, the biosurfactant produced by *L. acidophilus* was found to reduce the surface tension from 71.12 mN/m to 41.76 mN/m (Table 1).

**Table 1:** Different screening assays quantitative and qualitative results for the production of a biosurfactant from *L. acidophilus*

**Strain**	**Oil spreading test**	**Drop collapse test**	**%E24 (Olive oil)**	**%E24 (Petrol)**	**%E24 (Kerosene)**	**ST (mN/m)**
*L. acidophilus* MTCC-10307	Positive	Positive	68.70%	45.34%	35.68%	41.76

%E24: Emulsification index; ST: Surface tension.

**Figure 1.**
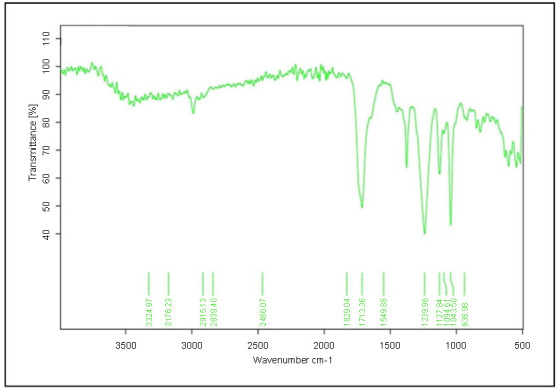
**Characterization of** *L. acidophilus***-derived biosurfactant via Fourier-transform infrared spectroscopy (FTIR)** **analysis.**

### Fourier-transform infrared spectroscopy analysis

The production and extraction of *L. acidophilus*-derived biosurfactant was performed in the MRS-Lac medium. The biosurfactant extracted from *L. acidophilus* was characterized by FTIR analysis. Understanding of the molecular composition of a biosurfactant is often crucial to exploring its potential uses in the future. As a result of FTIR analysis, it was possible to discover the chemical bonds existing in the biosurfactant and thereby to predict its chemical nature. An FTIR spectrometer is a fast and simple technology that can be used for the molecular characterization of biosurfactant. The FTIR spectrum of *L. acidophilus*-derived biosurfactant is presented in Figure 1. Considering the specific absorption peaks of the biosurfactant, it was concluded that its composition includes proteins, carbohydrates, and lipids. The important absorption peaks were at 1239–1127, 2915–1400, and 1549 cm^−1^, which correspond to carbohydrates, fats, and proteins, respectively.

### Antibacterial activity

**Figure 2.**
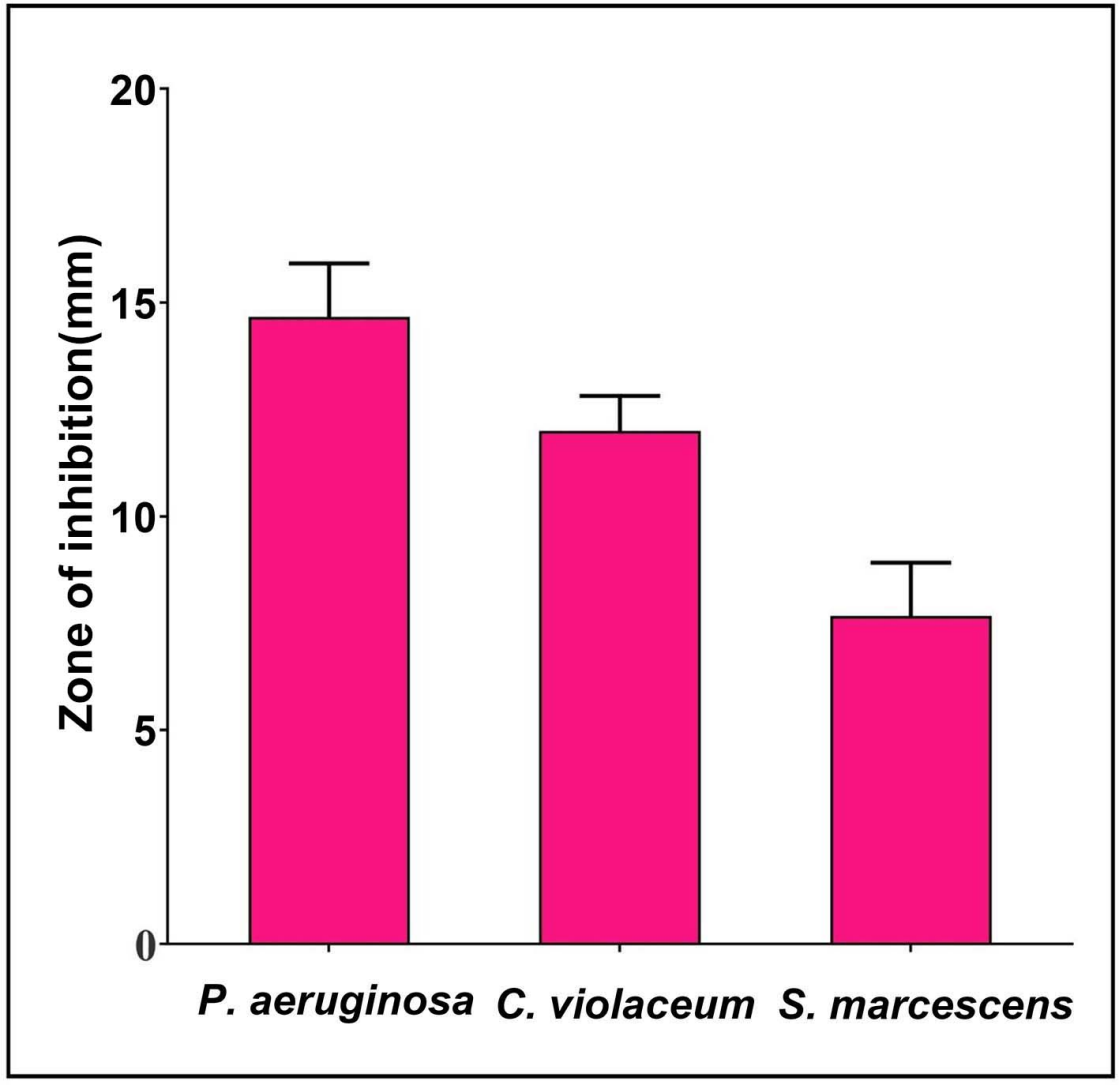
**Antibacterial activity of *L. acidophilus*-derived biosurfactant against different Gram-negative pathogenic bacteria.** Values are denoted as the mean ± SD of three independent experiments.

The antagonistic potential of *L. acidophilus* biosurfactant was studied by well diffusion/agar cup method against Gram-negative bacterial pathogens. Antibacterial activity results were presented in the form of zones of inhibition and demonstrated significant antagonistic activity against all bacterial test strains (Figure 2). Further, the crude *L. acidophilus* biosurfactant was found to have MIC values of 2.5 mg/mL against *C. violaceum* and *P. aeruginosa,* and 5 mg/mL against *S. marcescens*.

### Antibiofilm activity

**Figure 3.**
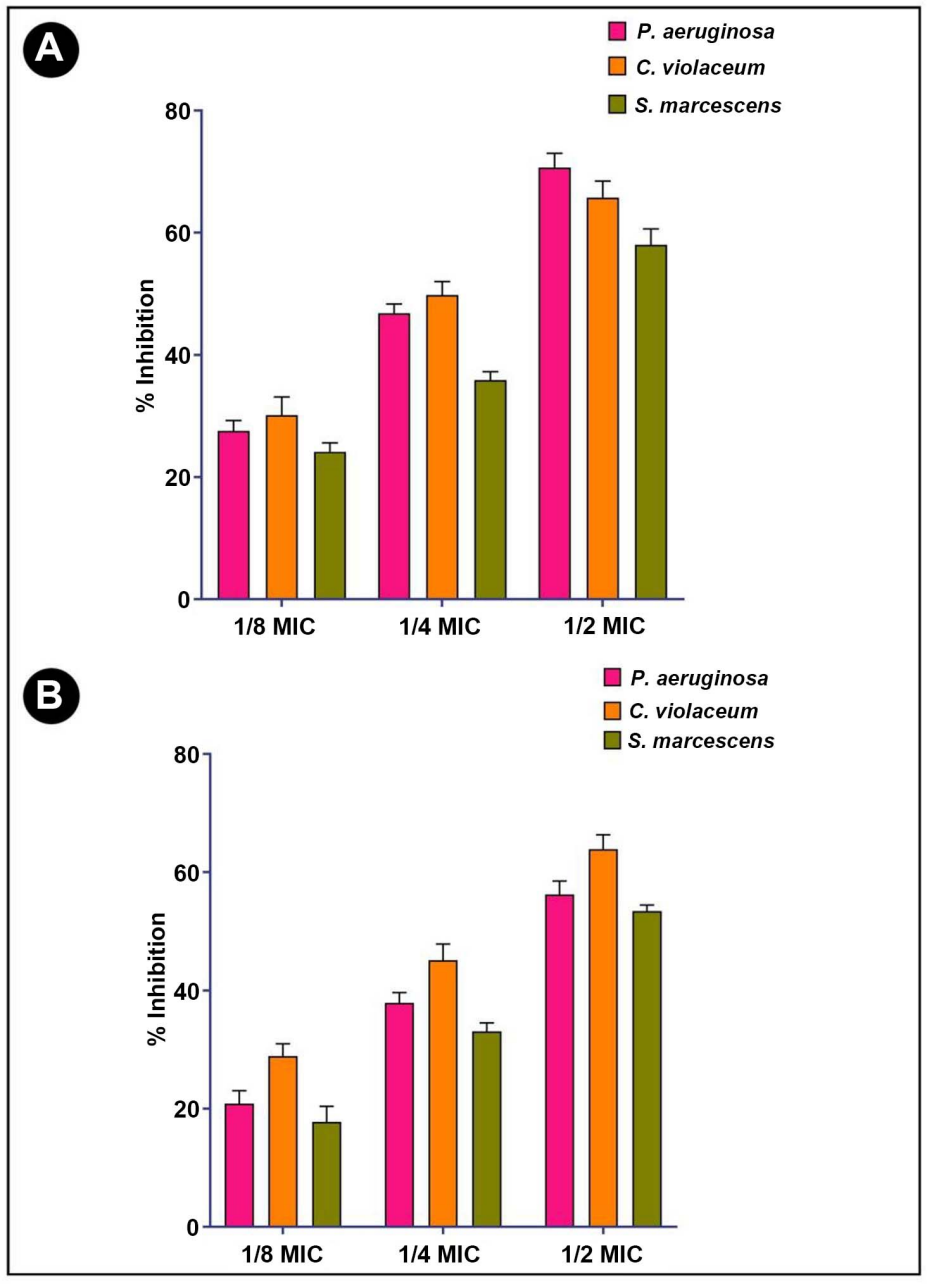
**Antibiofilm and EPS inhibition activity of *L. acidophilus-*derived biosurfactant against different Gram-negative pathogenic bacteria.** (A) Quantitative inhibition of biofilm production analysis using *L. acidophilus-*derived biosurfactant; (B) Quantitative inhibition of EPS production analysis using *L. acidophilus*-derived biosurfactant. Values are denoted as the mean ± SD of three independent experiments. MIC: Minimum inhibitory concentration; EPS: Exopolysaccharides.

A crystal violet assay was performed to determine the antibiofilm potential of *L. acidophilus* biosurfactant at sub-MIC concentrations against bacterial test strains. The obtained results indicated that the formation of biofilm was decreased with increasing biosurfactant concentration. The inhibition of biofilm formation at different sub-MIC concentrations is presented in Figure 3A.

### *L. acidophilus* biosurfactant inhibits exopolysaccharide production

In biofilms, EPS are biopolymers that are synthesized by bacteria and are embedded within the film. By retaining moisture within biofilm, the biopolymers of EPS form a matrix and hold it together, which helps to keep the cells together by keeping them moist. As a result of the treatment, it was also found that EPS production decreased in a concentration-dependent manner in all of the tested bacterial strains (Figure 3B).

### *L. acidophilus* biosurfactant disrupts the architecture of biofilm

**Figure 4.**
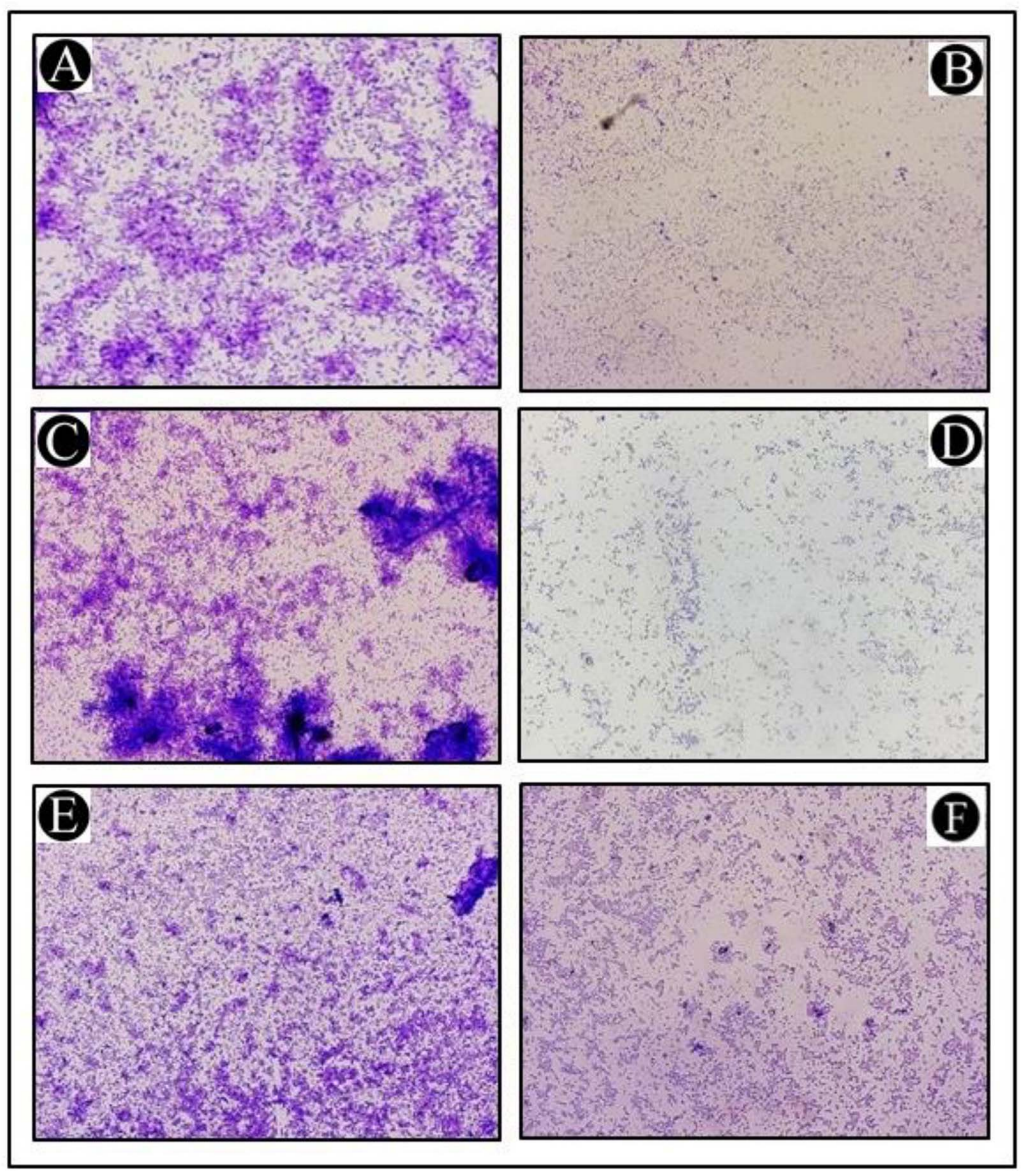
**Illustrative light micrograph of a biofilm showing the effects of *L. acidophilus*-derived biosurfactant at its highest sub-MICs.** (A) Control of *P. aeruginosa*; (B) Treatment of *P. aeruginosa* with 1/2 MIC; (C) Control of *C. violaceum*; (D) Treatment of *C. violaceum* with 1/2 MIC; (E) Control of *S. marcescens*; (F) Treatment of *S. marcescens* with 1/2 MIC. MIC: Minimum inhibitory concentration.

A topological analysis of the biofilm of all of the tested bacterial strains developed in the presence and absence of *L. acidophilus* biosurfactants was conducted by light microscopy. In the light microscopy analysis, the controls (normal biofilm developed) showed a well-grown biofilm, whereas treated samples showed dispersed bacterial cells (Figure 4A–4F).

### Effect of crude biosurfactant on quorum sensing-regulated virulence factors of *C. violaceum*

**Figure 5.**
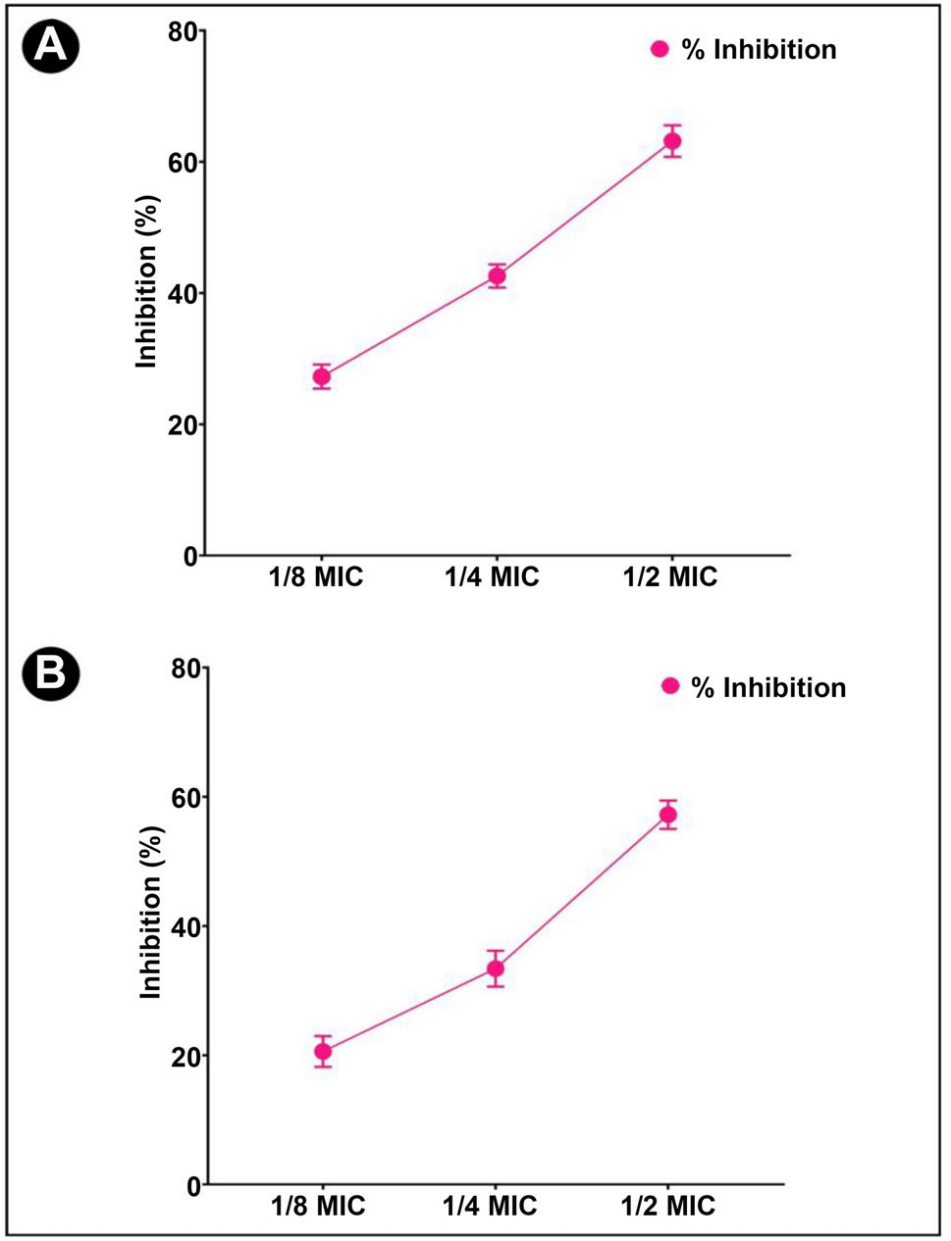
**Anti-QS activity of *L. acidophilus-*derived biosurfactant against *C. violaceum* and *S. marcescens*.** (A) Quantitative inhibition of violacein analysis in *C. violaceum* using *L. acidophilus*-derived biosurfactant; (B) Quantitative inhibition of prodigiosin analysis in *S. marcescens* using *L. acidophilus*-derived biosurfactant. Values are denoted as the mean ± SD of three independent experiments. QS: Quorum sensing; MIC: Minimum inhibitory concentration.

The *L. acidophilus* biosurfactant has been checked for its initial anti-QS activity by determining its effects on pigment production *by C. violaceum*, which is well-known to be QS-controlled. Reduced pigment production may serve as an indicator of the presence of anti-QS activity. Treatment with different sub-MIC concentrations in *C. violaceum* resulted in a reduction in the violacein synthesis by 63.17%, 42.61%, and 27.30%, respectively (Figure 5A). This evidently suggests that *L. acidophilus* biosurfactants are capable of exhibiting anti-QS activity.

### Effect of crude biosurfactant on quorum sensing-regulated virulence factors of *S. marcescens*

The *L. acidophilus* biosurfactant was also tested against *S. marcescens* for broad spectrum anti-QS activity. Prodigiosin, a pink-red pigment produced by *S. marcescens*, is regulated via QS. Figure 5B shows different sub-MIC concentrations of the *L. acidophilus* biosurfactant and was found to reduce the production of prodigiosin in *S. marcescens*. At these concentrations, inhibition of prodigiosin was found to be 57.24%, 33.41%, and 20.58%, respectively.

### Effect of crude biosurfactant on quorum sensing-regulated virulence factors of *P. aeruginosa*

**Figure 6.**
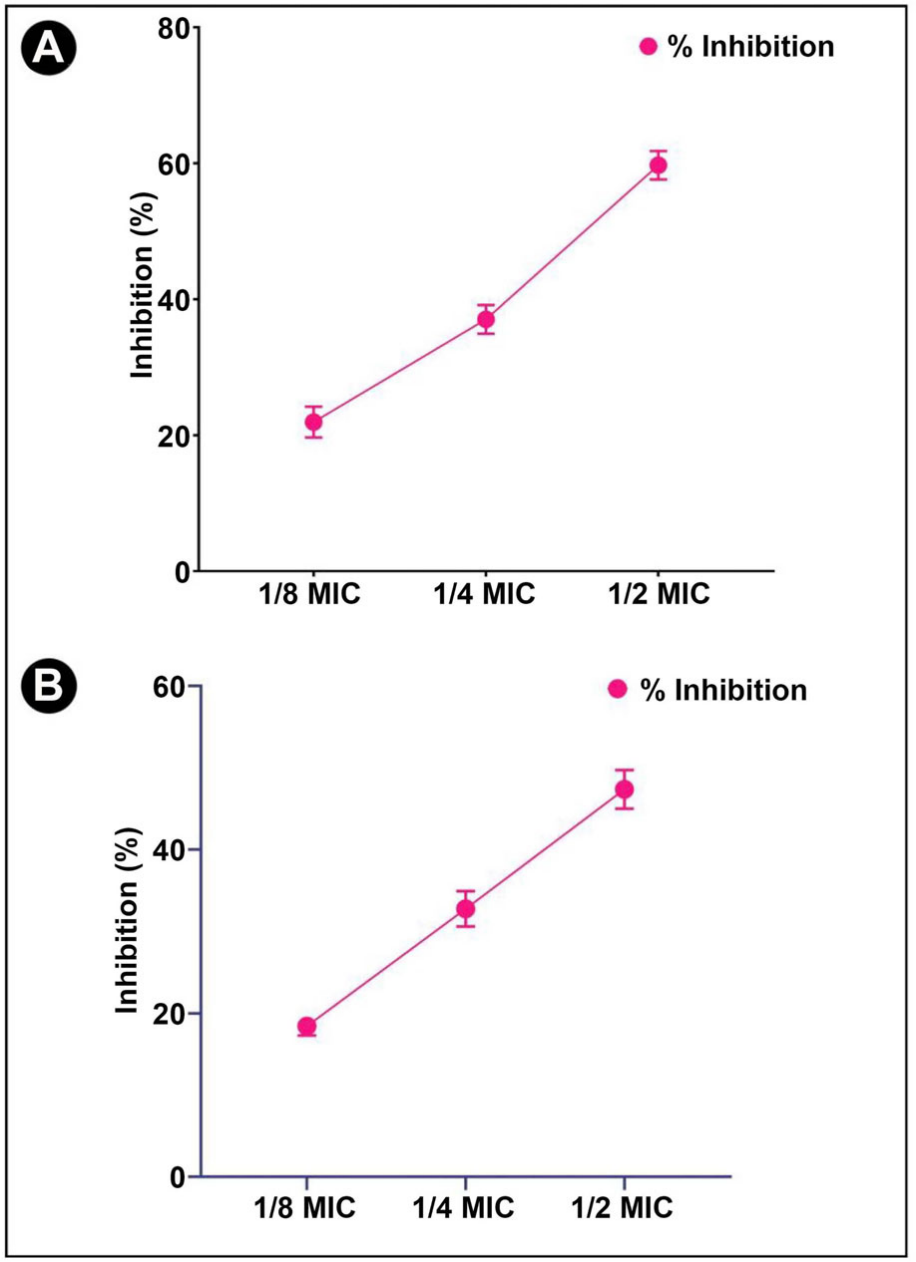
**Anti-QS activity of *L. acidophilus-*derived biosurfactant against *P. aeruginosa.*** (A) Quantitative inhibition of pyocyanin production analysis in *P. aeruginosa* using *L. acidophilus*-derived biosurfactant; (B) Quantitative inhibition of LasA protease production analysis in *P. aeruginosa* using *L. acidophilus*-derived biosurfactant against *P. aeruginosa*. Values are denoted as the mean ± SD of three independent experiments. QS: Quorum sensing; MIC: Minimum inhibitory concentration.

**Figure 7.**
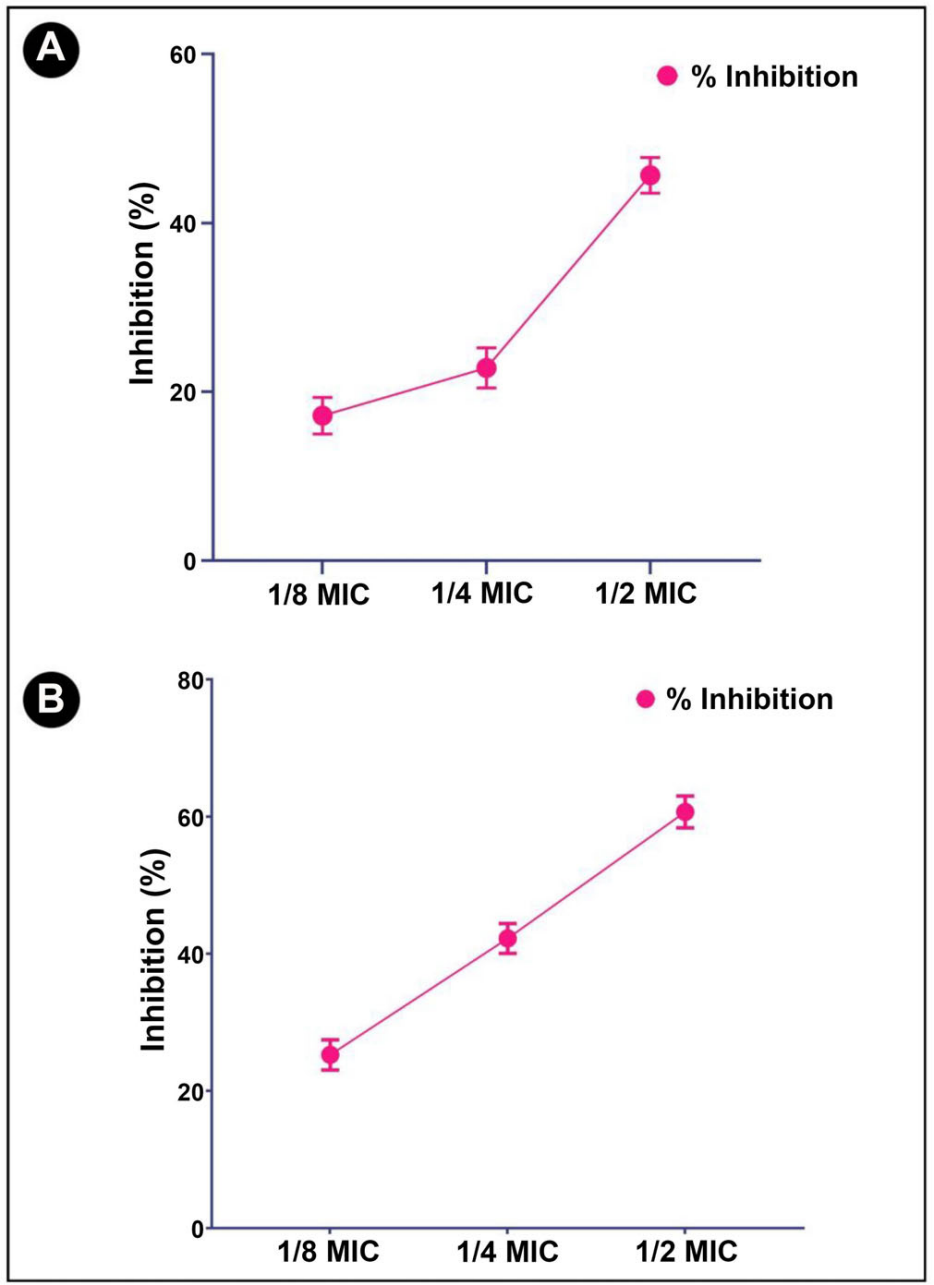
**Anti-QS activity of *L. acidophilus*-derived biosurfactant against *P. aeruginosa.*** (A) Quantitative inhibition of LasB protease production analysis in *P. aeruginosa* using *L. acidophilus*-derived biosurfactant; (B) Quantitative inhibition of total bacterial protease production analysis in *P. aeruginosa* using *L. acidophilus*-derived biosurfactant. Values are denoted as the mean ± SD of three independent experiments. QS: Quorum sensing; MIC: Minimum inhibitory concentration.

**Figure 8.**
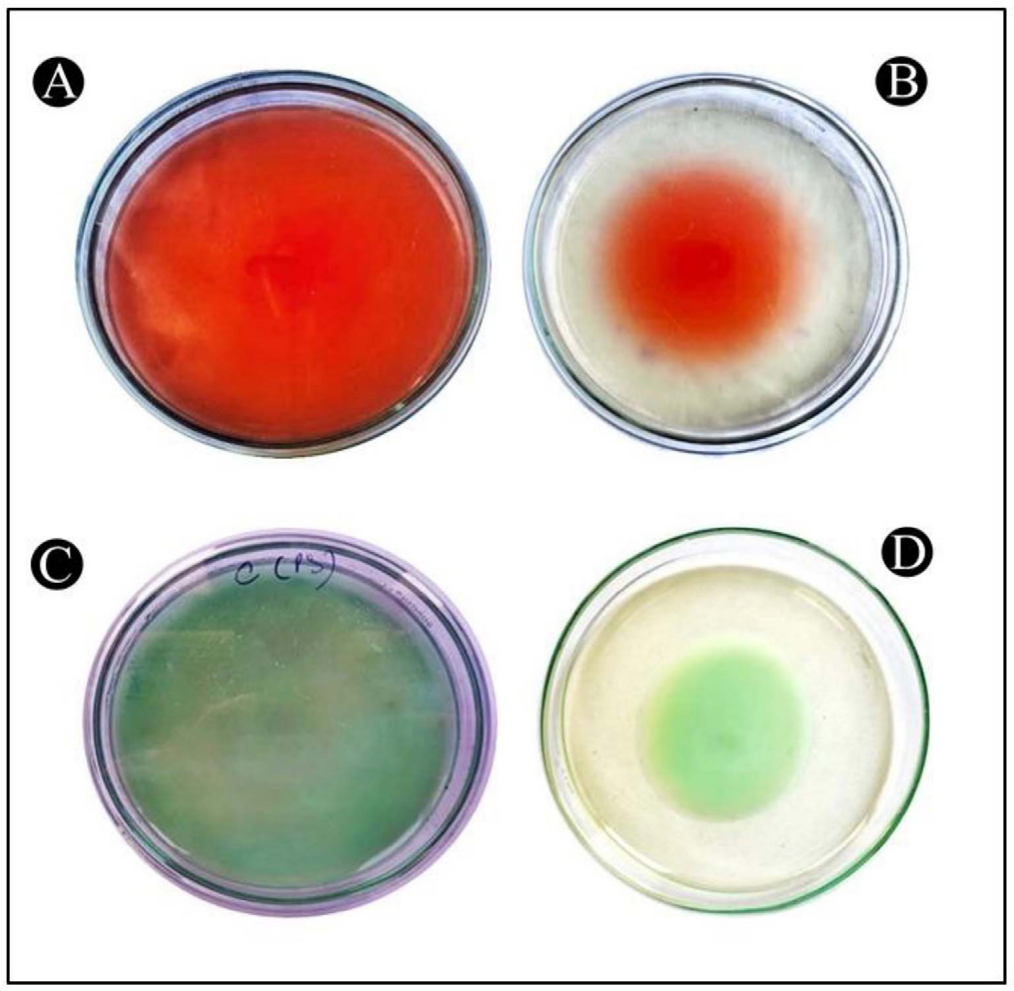
**Swimming motility inhibition of *P. aeruginosa* and *S. marcescens* by *L. acidophilus*-derived biosurfactant.** (A) Control of *P. aeruginosa*; (B) Treatment of *P. aeruginosa* with 1/2 MIC; (C) Control of *S. marcescens*; (D) Treatment of *S. marcescens* with 1/2 MIC. MIC: Minimum inhibitory concentration.

Further studies have been conducted on the crude biosurfactant to determine whether it has anti-QS potential toward *P. aeruginosa* via determining different virulent factors, such as LasB elastase, LasA protease, pyocyanin, and azocasein degrading protease activity. The potent virulent factor produced by *P. aeruginosa* is pyocyanin. As evident from the obtained results, the crude biosurfactant at sub-MIC concentrations was found to be effective in decreasing pyocyanin production in a dose-dependent manner (59.72%, 37.05%, and 21.93%, respectively) (Figure 6A). Based on the promising results obtained with pyocyanin, further tests were performed to check the inhibition of LasA protease activity. At sub-MIC concentrations of crude biosurfactant, LasA protease activity was reduced (47.36%, 32.76%, and 18.42%, respectively) (Figure 6B). Additionally, LasB elastase has the unique ability to cause necrotic skin lesions, corneal ulcers, and pulmonary hemorrhage, which makes it a very interesting and exceptional enzyme for studies. As a result of treatment with the crude biosurfactant, a significant reduction in LasB elastase activity was found (45.65%, 22.82%, and 17.36%, respectively) (Figure 7A). Moreover, a bacterial protease is a type of enzyme that cleaves proteins of the host cell (infected skin) and helps bacteria to invade and multiply. In the present study, it was also found that a crude biosurfactant also had the ability to suppress the production of bacterial proteases at sub-MIC concentrations (60.48%, 42.24%, and 25.62%, respectively) (Figure 7B). Swimming motility inhibition of * P. aeruginosa* and *S. marcescens* by *L. acidophilus*-derived biosurfactant can also be seen in Figure 8.

### Gas chromatography–mass spectrometry analysis

**Figure 9.**
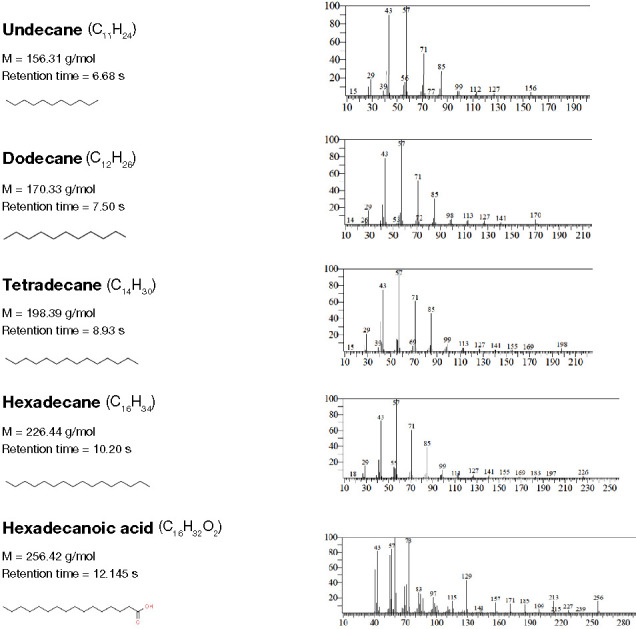
**Gas chromatography–mass spectrometry-based fatty acids profiles of biosurfactant derived from** *L. acidophilus.*

The extracted crude biosurfactant was analyzed by GC-MS analysis to determine the presence of compounds. GC-MS analysis revealed the presence of different fatty acids, such as undecane, dodecane, tetradecane, hexadecane, hexadecanoic acid and octadecanoic acid, and 2,3-dihydroxypropyl ester. The retention time, molecular formula, structure, and other details are presented in Figure 9.

**Figure 10.**
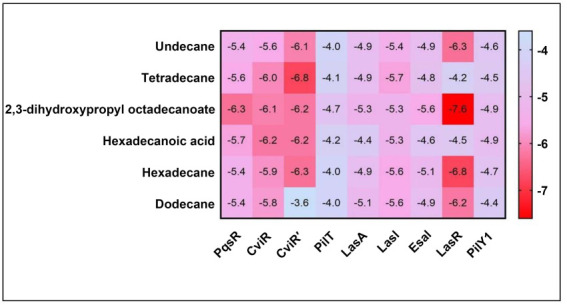
**Binding affinities of top-rated pose of ligand-receptor** **complex**.

**Figure 11.**
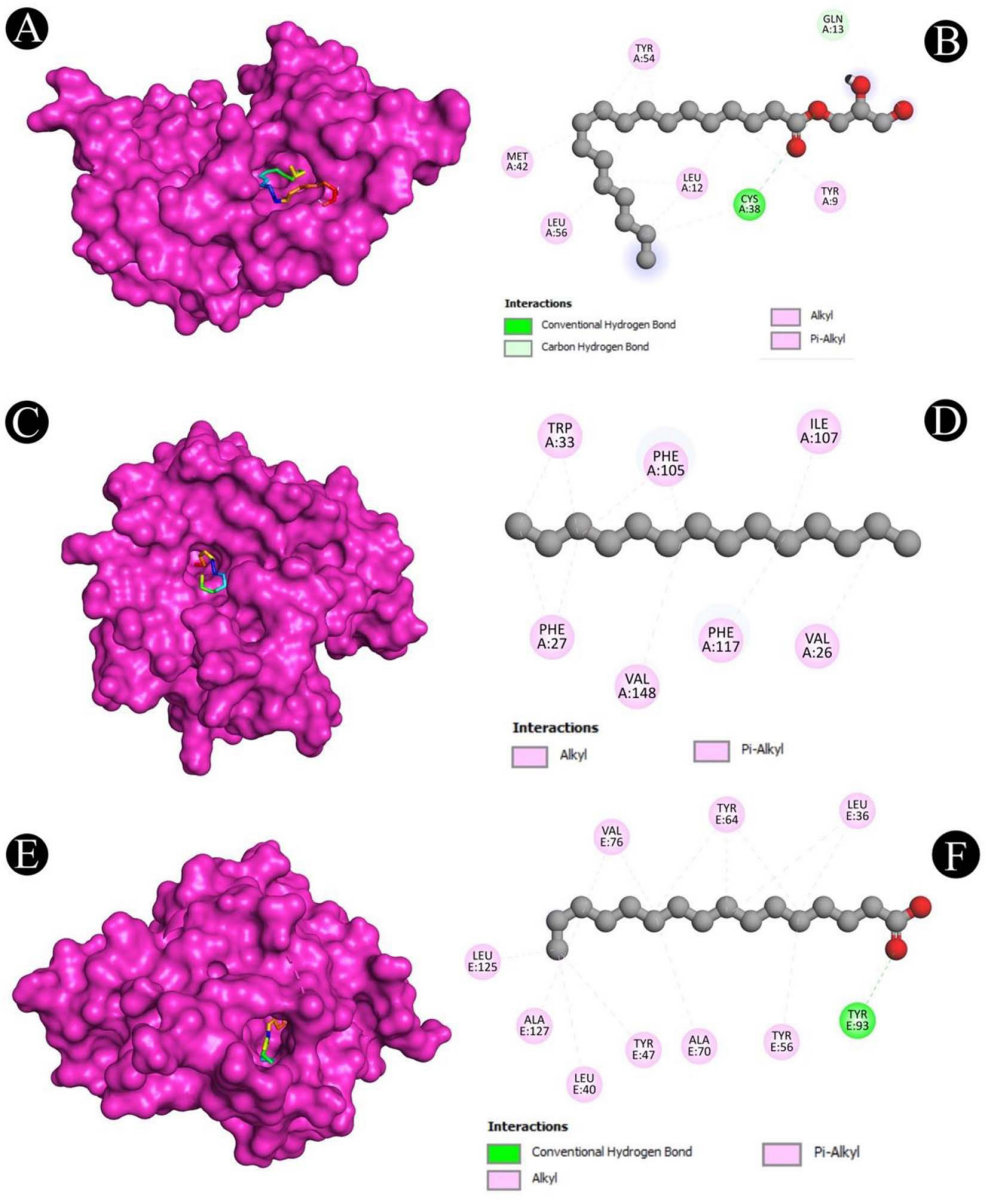
(A and B) Visualization of docking analysis of EsaI and 2,3-dihydroxypropyl octadecenoate; (C and D) Visualization of docking analysis of LasI and tetradecane; (E and F) Visualization of docking analysis of LasR and 2,3-dihydroxypropyl octadecanoate.

### Molecular docking analysis

**Figure 12.**
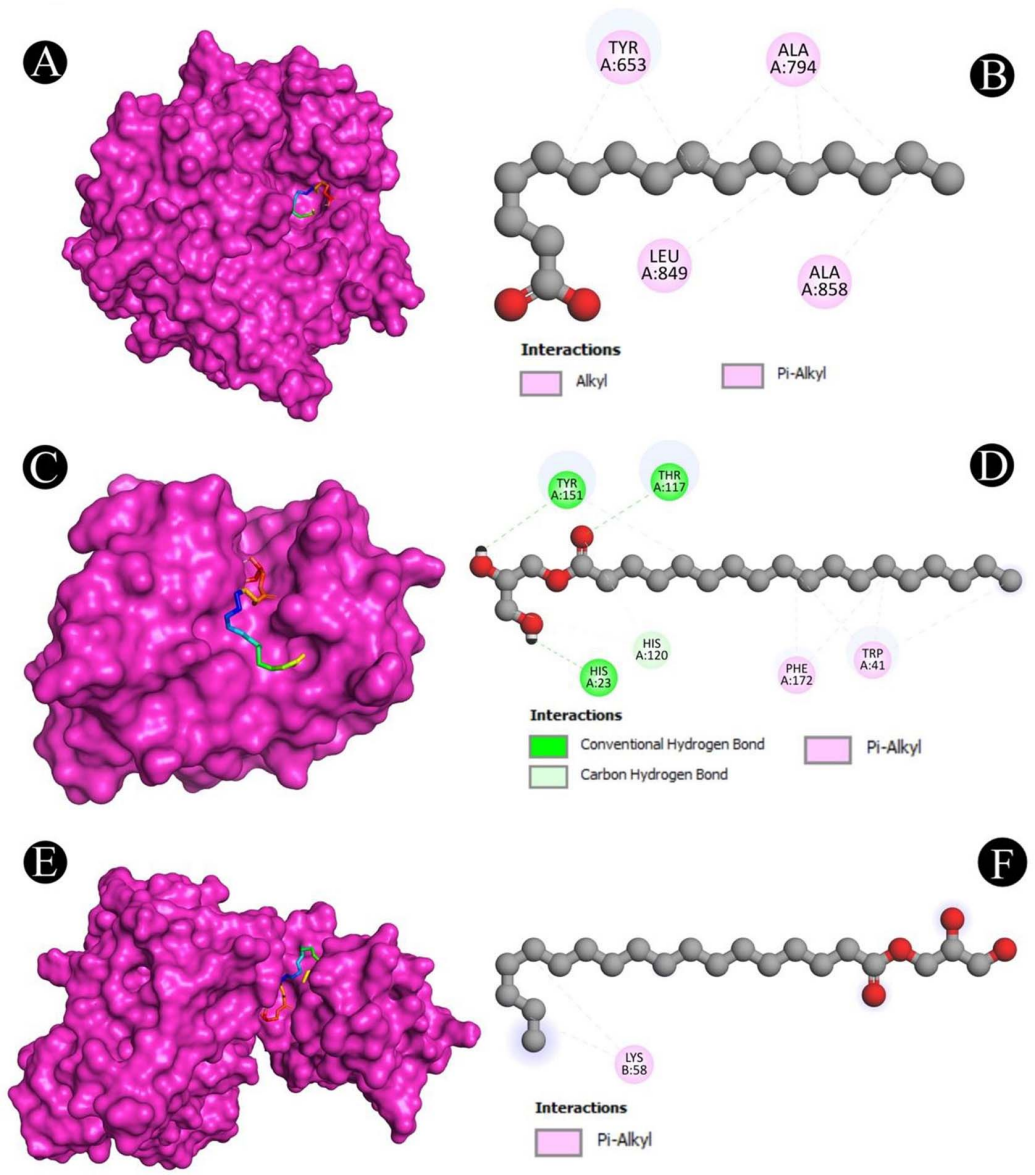
(A and B) Visualization of docking analysis of PilY1 and 2,3-dihydroxypropyl octadecenoate; (C and D) Visualization of docking analysis of LasA and 2,3-dihydroxypropyl octadecenoate; (E and F) Visualization of docking analysis of PilT and 2,3-dihydroxypropyl octadecanoate.

**Figure 13.**
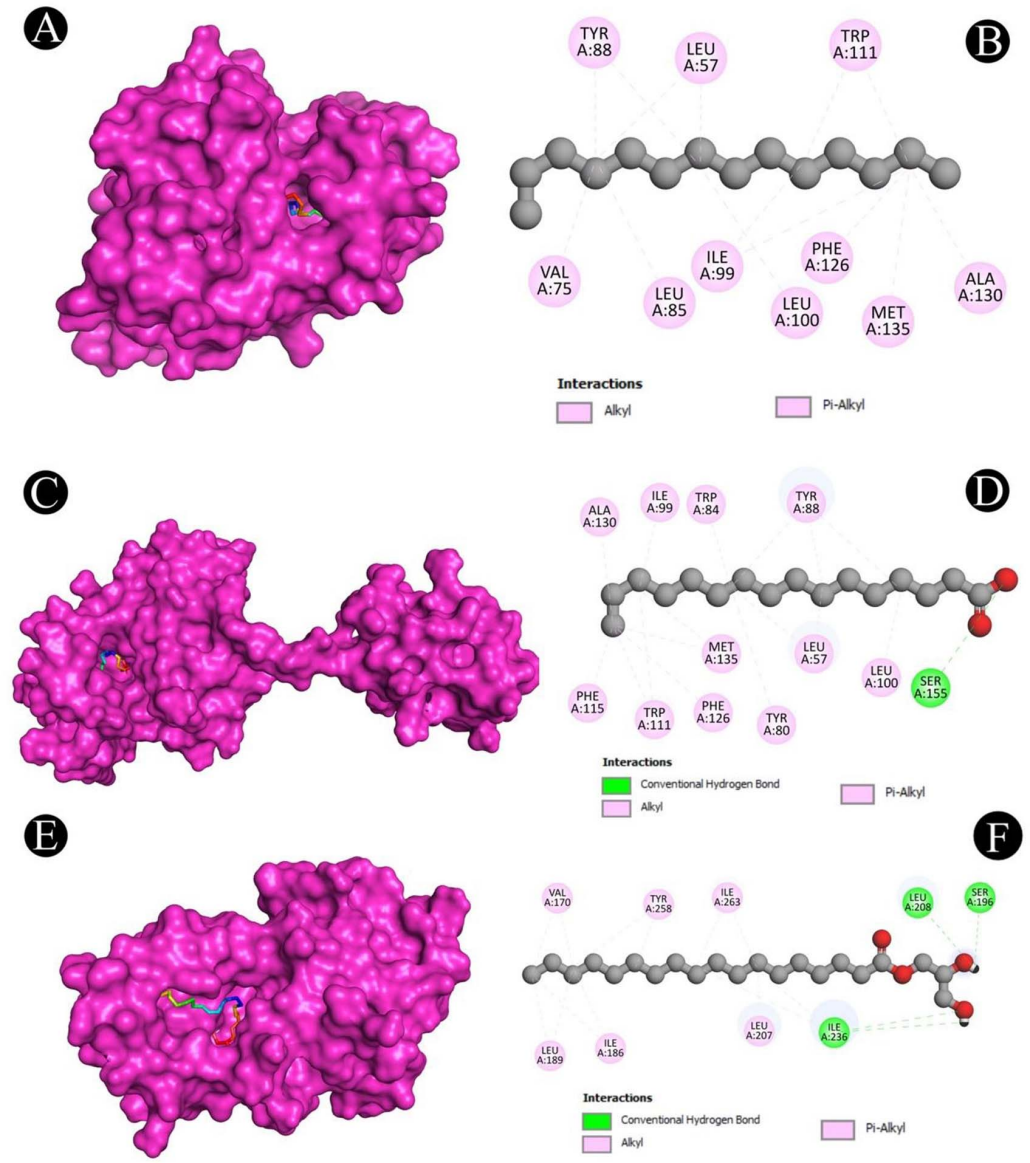
(A and B) Visualization of docking analysis of CViR’ and tetradecane; (C and D) Visualization of docking analysis of CViR and hexadecanoic acid; (E and F) Visualization of docking analysis of PqsR and 2,3-dihydroxypropyl octadecenoate.

Molecular docking with the QS and biofilm proteins was performed to gain a better understanding of the anti-virulence potential of the compounds identified from the crude biosurfactants. Different compounds identified from the crude biosurfactant exhibited different binding affinities. The binding energies are presented in Figure 10. The compounds with the highest binding energies toward respective proteins occupying the active site in various ways are shown in Figures 11–13 and Table 2. The results showed that tetradecane had the highest binding energy (−5.7 kcal/mol) toward LasI showing three alkyl bonds (VAL26, VAL148, and ILE107), seven pi-alkyl bonds (2*PHE27, 2*TRP33, 2*PHE105, and PHE117) and toward CviR’ (−6.8 kcal/mol) with nine alkyl bonds (VAL75, ALA130, 2*LEU57, LEU100, 2*ILE99, MET135, and LEU85), and seven pi-alkyl bonds (2*TYR88, 4*TRP111, and PHE126). On the other hand, 2,3-dihydroxypropyl octadecanoate had the highest binding energy (−5.6 kcal/mol) toward EsaI with one conventional hydrogen bond (CYS38), one carbon hydrogen bond (GLN13), and six alkyl bonds (CYS38, 3*LEU12, MET42, and LEU56); toward LasR (−7.6 kcal/mol) with one conventional hydrogen bond (TYR93) and eight alkyl bonds (ALA70, 2*VAL76, ALA127, 2*LEU36, LEU40, and LEU125); toward LasA (−5.3 kcal/mol) with three conventional hydrogen bonds (THR117, TYR151, and HIS23), two carbon hydrogen bonds (2*HIS120), and six pi-alkyl bond (3*TRP41, TYR151, and 2*PHE172); toward PqsR (−6.3 kcal/mol) with four conventional hydrogen bonds (2*ILE236, SER196, and LEU208), 11 alkyl bonds (2*VAL170, LEU207, 2*ILE236, 2*ILE263, ILE186, 2*LEU189, and ILE186), and two pi-alkyl bonds (2*TYR258); toward PilT (−4.7 kcal/mol) with two alkyl bonds (2*LYS58) and toward PilY1 (−4.9 kcal/mol) with five alkyl bonds (3*ALA794, ALA858, and LEU849) and two alkyl bonds (2*TYR653). Hexadecanoic acid had the highest binding energy (−6.1 kcal/mol) toward CviR with one conventional hydrogen bond (SER155), seven alkyl bonds (2*LEU57, ALA130, ILE99, 2*MET135, and LEU100), and 11 pi-alkyl bonds (TYR80, TRP84, 3*TYR88, 4*TRP111, PHE115, and PHE126).

## Discussion

Biofilm formation by different types of microbes is a global problem and is associated with drug resistance in microorganisms [40]. There is a general perception that biofilms are difficult to eradicate, and therefore natural products may offer a viable alternative. It has been reported that biosurfactants have potent antibiofilm potential [41]. When biosurfactants are present in lipid bilayer membranes, they can form pores and ion channels that disrupt their integrity and porosity. As a result, membranes are disrupted and cells die. Consequently, biosurfactants possess different types of biological functions, including antimycoplasma, antiviral, antifungal, and antibacterial [42, 43]. This leads us to hypothesize that crude biosurfactants derived from *L. acidophilus* may be effective against *P. aeruginosa* biofilms.

Initially, the biosurfactants production potential of *L. acidophilus* was qualitatively confirmed by drop collapse, oil displacement, emulsification assays, and surface tension measurements. In addition to being simple and effective, all these methods can be used for primary screening of the bacteria to confirm that the bacteria have the ability to produce biosurfactants [41]. Furthermore, the characterization of the extracted biosurfactants was performed using FTIR analysis. In FTIR analysis, major absorption peaks were found at 1239–1127, 2915–1400, and 1549 cm^−1^. In line with previous studies, this study yielded similar results and in line with Satpute et al. [44], which reported the presence of glycolipoproteins in biosurfactant from *L. acidophilus* as a result of FTIR characterization. A study conducted by Ghasemi et al. [45] showed that the biosurfactant derived from *L. rhamnosus* PTCC contained proteins and polysaccharides as multi-component mixture in FTIR analysis. The biosurfactant obtained from *L. rhamnosus* has also been shown to contain specific peaks of proteins and carbohydrates, as observed by Tahmourespour et al. [46].

Agar cup/well diffusion assay was used to test the antibacterial activity of crude biosurfactants extracted from *L. acidophilus* against different Gram-negative bacterial pathogens. Various properties, such as polarity, viscosity, etc., can influence a compound ability to diffuse in an agar plate, considerably affecting its antimicrobial activity [47]. Therefore, the MIC was determined as a way of overcoming the issue of solubility and diffusion in agar medium. Determination of the MIC of an antimicrobial agent is important because it provides information about the effectiveness of the drug against a particular microbe. MIC values are used to guide clinicians in selecting appropriate antimicrobial compound for treating infections, as well as to monitor the development of antimicrobial resistance [48]. MIC testing is also important in the development and testing of new antimicrobial agents, as it allows researchers to evaluate the potency and effectiveness of potential new drugs [49].

A variety of sub-MIC concentrations were then tested to determine their antibiofilm activity, as these concentrations do not impact the growth kinetics of the test organisms. The formation of bacterial biofilms can be modulated by sub-MIC doses [50]. These microbial biofilms play a key role in the survival of bacteria and in their ability to express virulence [33]. Consequently, successfully reducing biofilm formation can serve as a potential strategy for controlling the progression of a disease or eliminating a pathogen from an environment [51, 52]. This study was conducted in order to assess the antibiofilm properties of extracted biosurfactants against different Gram-negative bacterial pathogens in this context. Previously, a number of biosurfactants derived from different microbial sources have been reported to possess antibacterial and antibiofilm properties and have shown activity against a wide range of pathogenic bacteria [14–19]. In the present study, the antibiofilm potential of extracted biosurfactants was further evaluated to inhibit the established biofilm on glass coverslip surfaces of tested bacterial pathogens. Thus, the obtained results confirmed that the extracted biosurfactants exhibit significant antibiofilm activity when administered at sub-MIC concentrations.

It has been documented in the literature that microbe-mediated pathogenesis often occurs as a consequence of biofilm formation, as microorganisms within biofilms secrete several factors that enhance virulence [53]. When microorganisms invade a host, virulence factors play a critical role in initiating the invasion process. The QS system regulates violacein production by *C. violaceum* based on bacteria density. Although violacein is generally not considered a pathogenic factor, infections caused by *C. violaceum* can result in serious and even life-threatening complications in immunocompromised individuals [54]. In such cases, violacein contributes to the virulence of the bacteria and helps them evade the immune system. Despite the fact that cell-to-cell communication is essential for bacterial physiology and virulence, QS inhibitors have been shown to inhibit the production of violacein by *C. violaceum*, which suggests that QS inhibitors might be useful for the treatment of bacterial infections [54]. Prodigiosin, a bright red pigment produced by *S. marcescens*, is also synthesized via the QS. Prodigiosin is involved in the pathogenesis of *S. marcescens* infections, as it forms biofilms, exerts antimicrobial activity, modulates immune response, and induces cytotoxicity [55]. Thus, the regulation of QS activity can provide insight into infections caused by pathogenic bacteria.

Virulence factors secreted by *P. aeruginosa* can include several proteases, pyocyanin, elastase, etc., which play a significant role in facilitating disease progression [33]. Pyocyanin is a blue-green pigment produced by *P. aeruginosa* [56]. The cytotoxic activity of pyocyanin can be attributed to its ability to target a wide range of cellular processes and components, including the electron transport chain, vesicular transport, and the cell growth [56]. LasA elastase is a virulence factor produced by *P. aeruginosa* and its activity is regulated by the LasIR QS system. Elastase is a protease enzyme capable of degrading a variety of host proteins, including elastin, which is a major component of lung tissue [57]. When the bacterial population reaches a high density, the autoinducer molecule 3-oxo-C12-HSL, produced by LasI, binds to the transcription factor LasR, which activates the expression of the *lasA* gene, resulting in the production of LasA elastase. LasA elastase is considered a major virulence factor of *P. aeruginosa* and its activity has been associated with tissue damage and inflammation in the lungs of patients with cystic fibrosis and other respiratory infections. Inhibition of LasA elastase activity has been proposed as a potential therapeutic strategy to reduce the pathogenicity of *P. aeruginosa* infections [33]. LasB is another virulence factor produced by *P. aeruginosa*. Like LasA elastase, LasB is a protease enzyme that can degrade various host proteins, including elastin. The expression of LasB is also regulated by the LasIR QS system in *P. aeruginosa*. LasB is also considered as a major virulence factor of *P. aeruginosa* and has been associated with tissue damage, inflammation, and immune evasion in various infections caused by this bacterium [58]. In addition to its proteolytic activity, LasB also exhibits a variety of other functions, including disruption of cell membranes, stimulation of mucus production, and inhibition of host immune defenses. Hence, inhibition of LasB activity has been proposed as a potential therapeutic strategy to reduce the virulence of *P. aeruginosa* infections [59].

**Table 2:** Interactive active site residues top-rated pose of compounds with target proteins

**Serial number**	**Protein**	**Receptor–ligand**	**Interaction type**	**Distance**
1	PqsR-2,3-dihydroxypropyl octadecanoate	A:ILE236:HN–N:UNK1:O	Conventional hydrogen bond	2.50617
		N:UNK1:H–A:SER196:OG	Conventional hydrogen bond	2.69117
		N:UNK1:H–A:LEU208:O	Conventional hydrogen bond	2.66471
		N:UNK1:H–A:ILE236:O	Conventional hydrogen bond	2.59077
		N:UNK1:H–N:UNK1:O	Conventional hydrogen bond	1.95885
		A:VAL170–N:UNK1	Alkyl	5.02079
		N:UNK1–A:LEU207	Alkyl	5.49192
		N:UNK1–A:ILE236	Alkyl	4.95721
		N:UNK1–A:ILE263	Alkyl	4.4004
		N:UNK1–A:ILE236	Alkyl	3.99815
		N:UNK1–A:ILE263	Alkyl	5.16348
		N:UNK1–A:ILE186	Alkyl	4.71488
		N:UNK1–A:LEU189	Alkyl	5.15456
		N:UNK1:C–A:VAL170	Alkyl	3.83117
		N:UNK1:C–A:ILE186	Alkyl	4.37929
		N:UNK1:C–A:LEU189	Alkyl	4.7447
		A:TYR258–N:UNK1	Pi-Alkyl	4.14746
		A:TYR258–N:UNK1	Pi-Alkyl	4.54948
2	CviR-Hexadecanoic acid	A:SER155:OG–N:UNK1:O	Conventional hydrogen bond	3.06625
		A:LEU57–N:UNK1	Alkyl	5.3034
		A:ALA130–N:UNK1:C	Alkyl	4.09331
		N:UNK1–A:LEU57	Alkyl	4.95373
		N:UNK1–A:ILE99	Alkyl	5.08397
		N:UNK1–A:MET135	Alkyl	5.25806
		N:UNK1:C–A:MET135	Alkyl	4.72129
		N:UNK1–A:LEU100	Alkyl	4.83596
		A:TYR80–N:UNK1	Pi-Alkyl	4.22361
		A:TRP84–N:UNK1	Pi-Alkyl	4.68852
		A:TYR88–N:UNK1	Pi-Alkyl	4.69185
		A:TYR88–N:UNK1	Pi-Alkyl	4.82618
		A:TYR88–N:UNK1	Pi-Alkyl	4.41896
		A:TRP111–N:UNK1	Pi-Alkyl	4.92365
		A:TRP111–N:UNK1:C	Pi-Alkyl	4.97252
		A:TRP111–N:UNK1	Pi-Alkyl	4.18733
		A:TRP111–N:UNK1:C	Pi-Alkyl	4.32831
		A:PHE115–N:UNK1:C	Pi-Alkyl	4.96559
		A:PHE126–N:UNK1:C	Pi-Alkyl	4.97043
3	CviR’-Tetradecane	A:VAL75–N:UNK1	Alkyl	4.88709
		A:ALA130–N:UNK1	Alkyl	4.50512
		N:UNK1–A:LEU57	Alkyl	5.07901
		N:UNK1–A:LEU100	Alkyl	5.1371
		N:UNK1–A:ILE99	Alkyl	4.27644
		N:UNK1–A:ILE99	Alkyl	5.28038
		N:UNK1–A:MET135	Alkyl	5.33877
		N:UNK1–A:LEU57	Alkyl	4.76364
		N:UNK1–A:LEU85	Alkyl	4.91512
		A:TYR88–N:UNK1	Pi-Alkyl	4.86116
		A:TYR88–N:UNK1	Pi-Alkyl	4.03301
		A:TRP111–N:UNK1	Pi-Alkyl	5.24902
		A:TRP111–N:UNK1	Pi-Alkyl	5.22945
		A:TRP111–N:UNK1	Pi-Alkyl	4.47506
		A:TRP111–N:UNK1	Pi-Alkyl	4.09089
		A:PHE126–N:UNK1	Pi-Alkyl	5.10452
4	PilT-2,3-dihydroxypropyl octadecanoate	N:UNK1:H–N:UNK1:O	Conventional hydrogen bond	1.90383
		B:LYS58–N:UNK1	Alkyl	4.50049
		B:LYS58–N:UNK1	Alkyl	4.68472
5	LasA-2,3-dihydroxypropyl octadecanoate	A:THR117:OG1–N:UNK1:O	Conventional hydrogen bond	3.29146
		N:UNK1:H–A:TYR151:OH	Conventional hydrogen bond	2.48136
		N:UNK1:H–A:HIS23:NE2	Conventional hydrogen bond	2.7543
		A:HIS120:CE1–N:UNK1:O	Carbon hydrogen bond	3.70258
		N:UNK1:C–A:HIS120:NE2	Carbon hydrogen bond	3.52802
		A:TRP41–N:UNK1	Pi-Alkyl	4.9057
		A:TRP41–N:UNK1	Pi-Alkyl	5.2696
		A:TRP41–N:UNK1:C	Pi-Alkyl	5.15797
		A:TYR151–N:UNK1	Pi-Alkyl	4.19892
		A:PHE172–N:UNK1	Pi-Alkyl	4.91412
		A:PHE172–N:UNK1	Pi-Alkyl	4.71865
6	LasI-Tetradecane	A:VAL26–N:UNK1	Alkyl	4.60731
		A:VAL148–N:UNK1	Alkyl	5.01587
		N:UNK1–A:ILE107	Alkyl	4.46399
		A:PHE27–N:UNK1	Pi-Alkyl	5.37046
		A:PHE27–N:UNK1:C	Pi-Alkyl	5.0705
		A:TRP33–N:UNK1	Pi-Alkyl	4.9594
		A:TRP33–N:UNK1:C	Pi-Alkyl	4.61205
		A:PHE105–N:UNK1	Pi-Alkyl	5.30028
		A:PHE105–N:UNK1	Pi-Alkyl	4.27961
		A:PHE117–N:UNK1	Pi-Alkyl	5.10792
7	EsaI-2,3-dihydroxypropyl octadecanoate	A:CYS38:SG–N:UNK1:O	Conventional hydrogen bond	3.60805
		A:GLN13:CA–N:UNK1:O	Carbon hydrogen bond	3.61725
		A:CYS38–N:UNK1	Alkyl	4.1049
		N:UNK1–A:LEU12	Alkyl	4.61572
		N:UNK1–A:MET42	Alkyl	5.35796
		N:UNK1–A:LEU12	Alkyl	4.97704
		N:UNK1–A:LEU56	Alkyl	4.43872
		N:UNK1–A:LEU12	Alkyl	4.56324
		A:TYR9–N:UNK1	Pi-Alkyl	5.34577
		A:TYR54–N:UNK1	Pi-Alkyl	4.5584
		A:TYR54–N:UNK1	Pi-Alkyl	4.59545
		A:TYR54–N:UNK1	Pi-Alkyl	4.87913
8	LasR-2,3-dihydroxypropyl octadecanoate	E:TYR93:HH–N:UNK1:O	Conventional hydrogen bond	2.47142
		E:ALA70–N:UNK1	Alkyl	4.23715
		E:VAL76–N:UNK1	Alkyl	4.8954
		E:VAL76–N:UNK1	Alkyl	4.31626
		E:ALA127–N:UNK1	Alkyl	4.29468
		N:UNK1–E:LEU36	Alkyl	4.09764
		N:UNK1:C–E:LEU40	Alkyl	4.84962
		N:UNK1:C–E:LEU125	Alkyl	3.99152
		N:UNK1–E:LEU36	Alkyl	5.20163
		E:TYR47–N:UNK1:C	Pi-Alkyl	5.06921
		E:TYR56–N:UNK1	Pi-Alkyl	4.94303
		E:TYR64–N:UNK1	Pi-Alkyl	4.17073
		E:TYR64–N:UNK1	Pi-Alkyl	5.31868
		E:TYR64–N:UNK1	Pi-Alkyl	5.17785
9	PilY1-2,3-dihydroxypropyl octadecanoate	A:ALA794–N:UNK1	Alkyl	4.40422
		A:ALA794–N:UNK1	Alkyl	4.38667
		A:ALA794–N:UNK1	Alkyl	4.77119
		A:ALA858–N:UNK1	Alkyl	4.54833
		N:UNK1–A:LEU849	Alkyl	4.77259
		A:TYR653–N:UNK1	Pi-Alkyl	4.44572
		A:TYR653–N:UNK1	Pi-Alkyl	5.41154

In the present study, the crude biosurfactant extracted from *L. acidophilus* with different sub-MIC concentrations was compared for its effect on QS-associated proteins and factor activities. There is a good agreement between our results and those reported in the literature in terms of the inhibition of QS-associated proteins and virulence factors of *C. violaceum*, *S. marcescens*, and *P. aeruginosa*, although other natural products have also been reported to inhibit these factors and proteins to varying degrees (10% to 90%) [60]. Probiotic bacteria have been shown to inhibit formation of *P. aeruginosa* biofilm through the production of lactic acid by inhibiting the QS signal, specifically N-Acyl homoserine lactones (AHL) [61]. Similar inhibitory effects were also found in *L. casei*, *L. lactis*, and *L. helveticus* strains on *E. coli* O157:H7, *S. typhimurium*, and *L. monocytogenes* [62]. Furthermore, probiotics can produce organic acids that act as QS antagonists, inhibiting gene expression and preventing biofilm formation [63]. Furthermore, *L. brevis*, a strong probiotic, has also been shown to affect the QS system of pathogenic bacteria [60]. *L. plantarum* F-10 has been shown to have antimicrobial, antibiofilm, anti-QS, and antioxidant properties [64]. Similar to our investigation, another study has also investigated the antibiofilm effects of biosurfactants isolated from *L. casei* on *S. aureus* strains [65]. A number of strains of *L. plantarum*, *L. salivarius*, *L. casei*, and *L. reuteri* have been shown to inhibit the formation of biofilms and the expression of QS-related genes in *S. mutans* [66].

The present study further assessed other important factors that may contribute to the formation of biofilms in bacteria, such as EPS production and swarming motility, both of which were linked to the development of biofilms. QS-dependent EPS production is essential for the maturation of biofilms [67]. The crude biosurfactant interferes with QS, resulting in reduced EPS production. As such, it is believed that a crude biosurfactant dramatically reduces EPS and thus has the potential to minimize the level of resistance of the pathogen in its sessile state. The bacterial swarming motility tested was also found to be significantly decreased due to the treatment. Flagellar-driven motility is known to be effective for the development of biofilms by initiating surface attachment of the organism [68]. Therefore, swarming migration would be reduced if crude biosurfactants inhibited flagellar synthesis. As a result, crude biosurfactants were able to indirectly disrupt the QS system of bacteria, thereby impairing their ability to form biofilms.

Molecular docking analysis was further performed on compounds identified via GC-MS analysis to gain a better understanding of the effect of the compounds on the anti-virulence potential of the *L. acidophilus*-derived biosurfactant. Proteins involved in biofilm and QS were docked with the identified compounds. A number of studies have recently reported that fatty acids at lower concentration exhibit anti-hyphal, antibiofilm, anti-QS, and antifungal activities [69]. There are several compounds, for example, which have been found to selectively disrupt or inhibit biofilms of a number of pathogens, including *C. violaceum* [70], *S. aureus* [71], *P. aeruginosa* [72], and *C. albicans* [73, 74]. It has been shown that monounsaturated fatty acids inhibit the expression of several genes in *V. cholerae*, including palmitoleic and myristoleic acids [75, 76]. Their transcriptional regulators are also prevented from interacting with DNA by these molecules [77]. Furthermore, monounsaturated fatty acids can affect the expression of virulence factors, adhesion, and motility [78]. Other fatty acids are also reported to inhibit the biofilm formation and QS system of *A. baumannii* [79].

## Conclusion

As a result of the growing demand for eco-friendly materials, the use of biosurfactants has been increasing in a number of industrial sectors. The present study investigated the extraction and characterization of biosurfactant from the probiotic bacteria *L. acidophilus*. The extracted biosurfactant showed an antibacterial, antibiofilm, and anti-QS activity against different Gram-negative bacterial pathogens. In vitro studies have shown that the extracted biosurfactant inhibited the formation in the tested bacterial strains by its ability to decrease the swarming motility and its ability to regulate the virulence factors, such as pyocyanin, elastase, and protease. Accordingly, it can be suggested that the extracted biosurfactant intervenes in the QS system of bacterial pathogens and inhibits the production of virulent factors that contribute to the QS mechanism of the bacteria. As a therapeutic approach, it is important to target a QS system. This approach may be helpful in treating biofilm-related infections in an efficient manner. Therefore, the findings of the current study indicate that the extracted biosurfactant of *L. acidophilus* may be tested as an antibiofilm agent against a variety of Gram-negative bacteria in order to overcome the pathogenic processes associated with biofilms. However, in order to investigate potential pharmaceutical applications, a detailed study needs to be conducted.

## References

[1] Dzobo K (2022). The role of natural products as sources of therapeutic agents for innovative drug discovery. Compr Pharmacol.

[2] Yuan H, Ma Q, Ye L, Piao G (2016). The traditional medicine and modern medicine from natural products. Molecules.

[3] Azeem M, Hanif M, Mahmood K, Ameer N, Chughtai FRS, Abid U (2023). An insight into anticancer, antioxidant, antimicrobial, antidiabetic and anti-inflammatory effects of quercetin: a review. Polym Bull.

[4] Lu L, Hu W, Tian Z, Yuan D, Yi G, Zhou Y (2019). Developing natural products as potential anti-biofilm agents. Chin Med.

[5] Rutherford ST, Bassler BL (2012). Bacterial quorum sensing: its role in virulence and possibilities for its control. Cold Spring Harb Perspect Med.

[6] Lazar V (2011). Quorum sensing in biofilms–how to destroy the bacterial citadels or their cohesion/power?. Anaerobe.

[7] Schuster M, Greenberg EP (2007). Early activation of quorum sensing in *Pseudomonas aeruginosa* reveals the architecture of a complex regulon. BMC Genomics.

[8] Tuon FF, Dantas LR, Suss PH, Tasca Ribeiro VS (2022). Pathogenesis of the Pseudomonas aeruginosa biofilm: a review. Pathogens.

[9] Cassir N, Rolain J-M, Brouqui P (2014). A new strategy to fight antimicrobial resistance: the revival of old antibiotics. Front Microbiol.

[10] Gyawali R, Ibrahim SA (2014). Natural products as antimicrobial agents. Food Control.

[11] Ghasemi A, Moosavi-Nasab M, Setoodeh P, Mesbahi G, Yousefi G (2019). Biosurfactant production by lactic acid bacterium Pediococcus dextrinicus SHU1593 grown on different carbon sources: strain screening followed by product characterization. Sci Rep.

[12] Santos DKF, Rufino RD, Luna JM, Santos VA, Sarubbo LA (2016). Biosurfactants: multifunctional biomolecules of the 21st century. Int J Mol Sci.

[13] Reid G, Younes JA, Van der Mei HC, Gloor GB, Knight R, Busscher HJ (2011). Microbiota restoration: natural and supplemented recovery of human microbial communities. Nat Rev Microbiol.

[14] Adnan M, Siddiqui AJ, Hamadou WS, Ashraf SA, Hassan MI, Snoussi M (2021). Functional and structural characterization of pediococcus pentosaceus-derived biosurfactant and its biomedical potential against bacterial adhesion, quorum sensing, and biofilm formation. Antibiotics.

[15] Banat IM, Makkar RS, Cameotra SS (2000). Potential commercial applications of microbial surfactants. Appl Microbiol Biotechnol.

[16] Jahan R, Bodratti AM, Tsianou M, Alexandridis P (2020). Biosurfactants, natural alternatives to synthetic surfactants: physicochemical properties and applications. Adv Colloid Interface Sci.

[17] Patel M, Siddiqui AJ, Ashraf SA, Surti M, Awadelkareem AM, Snoussi M (2022). Lactiplantibacillus plantarum-derived biosurfactant attenuates quorum sensing-mediated virulence and biofilm formation in Pseudomonas aeruginosa and Chromobacterium violaceum. Microorganisms.

[18] Patel M, Siddiqui AJ, Hamadou WS, Surti M, Awadelkareem AM, Ashraf SA (2021). Inhibition of bacterial adhesion and antibiofilm activities of a glycolipid biosurfactant from Lactobacillus rhamnosus with its physicochemical and functional properties. Antibiotics.

[19] Seydlová G, Svobodová J (2008). Review of surfactin chemical properties and the potential biomedical applications. Central Eur J Med.

[20] Joe MM, Gomathi R, Benson A, Shalini D, Rengasamy P, Henry AJ (2019). Simultaneous application of biosurfactant and bioaugmentation with rhamnolipid-producing shewanella for enhanced bioremediation of oil-polluted soil. Appl Sci.

[21] Płaza GA, Zjawiony I, Banat IM (2006). Use of different methods for detection of thermophilic biosurfactant-producing bacteria from hydrocarbon-contaminated and bioremediated soils. J Petrol Sci Eng.

[22] Satpute SK, Banpurkar AG, Dhakephalkar PK, Banat IM, Chopade BA (2010). Methods for investigating biosurfactants and bioemulsifiers: a review. Crit Rev Biotechnol.

[23] Martins PC, Martins VG (2018). Biosurfactant production from industrial wastes with potential remove of insoluble paint. Int Biodeterior Biodegrad.

[24] George S, Jayachandran K (2009). Analysis of rhamnolipid biosurfactants produced through submerged fermentation using orange fruit peelings as sole carbon source. Appl Biochem Biotechnol.

[25] Ferreira A, Vecino X, Ferreira D, Cruz J, Moldes A, Rodrigues L (2017). Novel cosmetic formulations containing a biosurfactant from Lactobacillus paracasei. Colloids Surf B Biointerfaces.

[26] Adnan M, Patel M, Deshpande S, Alreshidi M, Siddiqui AJ, Reddy MN (2020). Effect of *Adiantum philippense* extract on biofilm formation, adhesion with its antibacterial activities against foodborne pathogens, and characterization of bioactive metabolites: an *in vitro-in silico* approach. Front Microbiol.

[27] Wayne P (2010). Clinical and Laboratory Standards Institute: performance standards for antimicrobial susceptibility testing: 20th informational supplement. CLSI document M100-S20..

[28] Ghaima KK, Rasheed SF, Ahmed EF (2013). Antibiofilm, antibacterial and antioxidant activities of water extract of Calendula officinalis flowers. Int J Biol Pharm Res.

[29] Luft JH (1971). Ruthenium red and violet. II. Fine structural localization in animal tissues. Anat Rec.

[30] Musthafa KS, Ravi AV, Annapoorani A, Packiavathy ISV, Pandian SK (2010). Evaluation of anti-quorum-sensing activity of edible plants and fruits through inhibition of the N-acyl-homoserine lactone system in Chromobacterium violaceum and Pseudomonas aeruginosa. Chemotherapy.

[31] Matz C, Deines P, Boenigk J, Arndt H, Eberl L, Kjelleberg S (2004). Impact of violacein-producing bacteria on survival and feeding of bacterivorous nanoflagellates. Appl Environ Microbiol.

[32] Slater H, Crow M, Everson L, Salmond GP (2003). Phosphate availability regulates biosynthesis of two antibiotics, prodigiosin and carbapenem, in Serratia via both quorum-sensing-dependent and-independent pathways. MolMicrobiol.

[33] Ugurlu A, Yagci AK, Ulusoy S, Aksu B, Bosgelmez-Tinaz G (2016). Phenolic compounds affect production of pyocyanin, swarming motility and biofilm formation of *Pseudomonas aeruginosa*. Asian–Pac J Trop Biomed.

[34] Kessler E, Safrin M, Olson JC, Ohman DE (1993). Secreted LasA of Pseudomonas aeruginosa is a staphylolytic protease. J Biol Chem.

[35] Adonizio A, Kong K-F, Mathee K (2008). Inhibition of quorum sensing-controlled virulence factor production in Pseudomonas aeruginosa by South Florida plant extracts. Antimicrob Agents Chemother.

[36] Packiavathy IASV, Priya S, Pandian SK, Ravi AV (2014). Inhibition of biofilm development of uropathogens by curcumin—an anti-quorum sensing agent from *Curcuma longa*. Food Chem.

[37] Morris GM, Huey R, Olson AJ (2008). Using AutoDock for ligand-receptor docking. Curr Protoc Bioinform.

[38] Studio D. 2.1; Accelrys Inc.

[39] DeLano WL (2002). Pymol: an open-source molecular graphics tool. CCP4 Newsl Protein Crystallogr.

[40] Parsek MR, Singh PK (2003). Bacterial biofilms: an emerging link to disease pathogenesis. Annu Rev Microbiol.

[41] Singh P, Cameotra SS (2004). Potential applications of microbial surfactants in biomedical sciences. Trends Biotechnol.

[42] Fracchia L, Cavallo M, Martinotti MG, Banat IM (2012). Biosurfactants and bioemulsifiers biomedical and related applications–present status and future potentials. Biomed Sci Eng Technol.

[43] Kracht M, Rokos H, Özel M, Kowall M, Pauli G, Vater J (1999). Antiviral and hemolytic activities of surfactin isoforms and their methyl ester derivatives. J Antibiot.

[44] Satpute SK, Mone NS, Das P, Banpurkar AG, Banat IM (2018). Lactobacillus *acidophilus* derived biosurfactant as a biofilm inhibitor: a promising investigation using microfluidic approach. Appl Sci.

[45] Ghasemi A, Moosavi-Nasab M, Behzadnia A, Rezaei M (2018). Enhanced biosurfactant production with low-quality date syrup by *Lactobacillus rhamnosus* using a fed-batch fermentation. Food Sci Biotechnol.

[46] Tahmourespour A, Kasra-Kermanshahi R, Salehi R (2019). *Lactobacillus rhamnosus* biosurfactant inhibits biofilm formation and gene expression of caries-inducing *Streptococcusmutans*. Dental Res J.

[47] Balouiri M, Sadiki M, Ibnsouda SK (2016). Methods for *in vitro* evaluating antimicrobial activity: a review. J Pharm Anal.

[48] Saritha K, Rajesh A, Manjulatha K, Setty OH, Yenugu S (2015). Mechanism of antibacterial action of the alcoholic extracts of Hemidesmus indicus (L.) R. Br. ex Schult, Leucas aspera (Wild.), Plumbago zeylanica L., and Tridax procumbens (L.) R. Br. ex Schult. Front Microbiol.

[49] Foerster S, Unemo M, Hathaway LJ, Low N, Althaus CL (2016). Time-kill curve analysis and pharmacodynamic modelling for in vitro evaluation of antimicrobials against Neisseria gonorrhoeae. BMC Microbiol.

[50] Das A, Das MC, Sandhu P, Das N, Tribedi P, De UC (2017). Antibiofilm activity of Parkia javanica against Pseudomonas aeruginosa: a study with fruit extract. RSC Adv.

[51] Kumar L, Chhibber S, Harjai K (2013). Zingerone inhibit biofilm formation and improve antibiofilm efficacy of ciprofloxacin against Pseudomonas aeruginosa PAO1. Fitoterapia.

[52] Sarkar R, Chaudhary SK, Sharma A, Yadav KK, Nema NK, Sekhoacha M (2014). Anti-biofilm activity of Marula—a study with the standardized bark extract. J Ethnopharmacol.

[53] Williams P (2007). Quorum sensing, communication and cross-kingdom signalling in the bacterial world. Microbiology.

[54] Abraham W-R (2005). Controlling pathogenic gram-negative bacteria by interfering with their biofilm formation. Drug Des Rev.

[55] Kothari V, Sharma S, Padia D (2017). Recent research advances on *Chromobacterium violaceum*. Asian–Pac J Trop Med.

[56] Essar DW, Eberly L, Hadero A, Crawford I (1990). Identification and characterization of genes for a second anthranilate synthase in *Pseudomonas aeruginosa*: interchangeability of the two anthranilate synthases and evolutionary implications. J Bacteriol.

[57] Ahmed SA, Rudden M, Smyth TJ, Dooley JS, Marchant R, Banat IM (2019). Natural quorum sensing inhibitors effectively downregulate gene expression of Pseudomonas aeruginosa virulence factors. Appl Microbiol Biotechnol.

[58] Cathcart GRA, Quinn D, Greer B, Harriott P, Lynas JF, Gilmore BF (2011). Novel inhibitors of the Pseudomonas aeruginosa virulence factor LasB: a potential therapeutic approach for the attenuation of virulence mechanisms in pseudomonal infection. Antimicrob Agents Chemother.

[59] Kaya C, Walter I, Alhayek A, Shafiei R, Jézéquel G, Andreas A (2022). Structure-based design of α-substituted mercaptoacetamides as inhibitors of the virulence factor LasB from *Pseudomonas aeruginosa*. ACS Infect Dis.

[60] Li Q, Pan Y, Ding L, Hong H, Yan S, Wu B (2017). Draft genome sequence of *Lactobacillus brevis* Strain 3M004, a probiotic with potential quorum-sensing regulation. Genome Announc.

[61] Kiymaci ME, Altanlar N, Gumustas M, Ozkan SA, Akin A (2018). Quorum sensing signals and related virulence inhibition of Pseudomonas aeruginosa by a potential probiotic strain’s organic acid. Microb Pathog.

[62] Gómez NC, Ramiro JM, Quecan BX, de Melo Franco BD (2016). Use of potential probiotic lactic acid bacteria (LAB) biofilms for the control of Listeria monocytogenes, Salmonella Typhimurium, and Escherichia coli O157:H7 biofilms formation. Front Microbiol.

[63] Barzegari A, Kheyrolahzadeh K, Hosseiniyan Khatibi S, Sharifi S, Memar M, Zununi Vahed S (2020). The battle of probiotics and their derivatives against biofilms. Infect Drug Resist 13:659–72..

[64] Onbas T, Osmanagaoglu O, Kiran F (2019). Potential properties of Lactobacillus plantarum F-10 as a bio-control strategy for wound infections. Probiotics Antimicrob Proteins.

[65] Merghni A, Dallel I, Noumi E, Kadmi Y, Hentati H, Tobji S (2017). Antioxidant and antiproliferative potential of biosurfactants isolated from Lactobacillus casei and their anti-biofilm effect in oral Staphylococcus aureus strains. Microb Pathog.

[66] Wasfi R, Abd El-Rahman OA, Zafer MM, Ashour HM (2018). Probiotic Lactobacillus sp. inhibit growth, biofilm formation and gene expression of caries-inducing Streptococcus mutans. J Cell Mol Med.

[67] Watnick PI, Kolter R (1999). Steps in the development of a Vibrio cholerae El Tor biofilm. Mol Microbiol.

[68] Pratt LA, Kolter R (1998). Genetic analysis of Escherichia coli biofilm formation: roles of flagella, motility, chemotaxis and type I pili. Mol Microbiol.

[69] Kumar P, Lee J-H, Beyenal H, Lee J (2020). Fatty acids as antibiofilm and antivirulence agents. Trends Microbiol.

[70] Davies DG, Marques CN (2009). A fatty acid messenger is responsible for inducing dispersion in microbial biofilms. J Bacteriol.

[71] Kim Y-G, Lee J-H, Raorane CJ, Oh ST, Park JG, Lee J (2018). Herring oil and omega fatty acids inhibit Staphylococcus aureus biofilm formation and virulence. Front Microbiol.

[72] Wenderska IB, Chong M, McNulty J, Wright GD, Burrows LL (2011). Palmitoyl-DL-carnitine is a multitarget inhibitor of Pseudomonas aeruginosa biofilm development. ChemBioChem.

[73] Cui C, Song S, Yang C, Sun X, Huang Y, Li K (2019). Disruption of quorum sensing and virulence in *Burkholderia cenocepacia* by a structural analogue of the cis-2-dodecenoic acid signal. Appl Environ Microbiol.

[74] Prasath KG, Sethupathy S, Pandian SK (2019). Proteomic analysis uncovers the modulation of ergosterol, sphingolipid and oxidative stress pathway by myristic acid impeding biofilm and virulence in Candida albicans. J Proteomics.

[75] Childers BM, Cao X, Weber GG, Demeler B, Hart PJ, Klose KE (2011). N-terminal residues of the Vibrio cholerae virulence regulatory protein ToxT involved in dimerization and modulation by fatty acids. J Biol Chem.

[76] Lowden MJ, Skorupski K, Pellegrini M, Chiorazzo MG, Taylor RK, Kull FJ (2010). Structure of Vibrio cholerae ToxT reveals a mechanism for fatty acid regulation of virulence genes. Proc Nat Acad Sci.

[77] Plecha SC, Withey JH (2015). Mechanism for inhibition of Vibrio cholerae ToxT activity by the unsaturated fatty acid components of bile. J Bacteriol.

[78] Kabara JJ, Swieczkowski DM, Conley AJ, Truant JP (1972). Fatty acids and derivatives as antimicrobial agents. Antimicrob Agents Chemother.

[79] Nicol M, Alexandre S, Luizet J-B, Skogman M, Jouenne T, Salcedo SP (2018). Unsaturated fatty acids affect quorum sensing communication system and inhibit motility and biofilm formation of Acinetobacter baumannii. Int J Mol Sci.

